# Supplementary material for: Detecting and quantifying heterogeneity in susceptibility using contact tracing data
Source: PLoS Comput Biol. 2024 Jul 29;20(7):e1012310. doi: 10.1371/journal.pcbi.1012310 (PMC11309420; doi:10.1371/journal.pcbi.1012310)
Supplement: S1 Text — (PDF) [file pcbi.1012310.s001.pdf]

Supporting Information S1: Derivation of C_d

Beth M. Tuschhoff, David A. Kennedy

Department of Biology, The Pennsylvania State University, University Park, Pennsylvania, United States of America

---

The coefficient of variation is defined as standard deviation divided by the mean. Hence, C_d is the standard deviation of risk divided by the mean risk.

The mean risk, μ_r, is given by

$$\mu_r = E(\text{risk}) = r_A f_A + r_B(1 - f_A),$$

and the standard deviation of risk, σ_r, is given by

$$\begin{aligned} \sigma_r &= \sqrt{{\sigma_r}^2} \\ &= \sqrt{E(\text{risk}^2) - E(\text{risk})^2} \\ &= \sqrt{({r_A}^2 f_A + {r_B}^2(1 - f_A)) - (r_A f_A + r_B(1 - f_A))^2}. \end{aligned}$$

$C_d = \frac{\sigma_r}{\mu_r}$ can then be simplified to

$$C_d = \frac{(r_A - r_B)\sqrt{f_A(1 - f_A)}}{r_A f_A + r_B(1 - f_A)}.$$
