# Supplementary material for: Detecting and quantifying heterogeneity in susceptibility using contact tracing data
Source: PLoS Comput Biol. 2024 Jul 29;20(7):e1012310. doi: 10.1371/journal.pcbi.1012310 (PMC11309420; doi:10.1371/journal.pcbi.1012310)
Supplement: S2 Text — (PDF) [file pcbi.1012310.s002.pdf]

# Supporting Information S2: Changing $N$

Beth M. Tuschhoff, David A. Kennedy

*Department of Biology, The Pennsylvania State University, University Park, Pennsylvania, United States of America*

---

---

For both underlying models, we explored the effect of changing the number of individuals in each contact network $N$ on our power to detect heterogeneity in susceptibility and our ability to predict SIR dynamics. In Supporting Information S11, we further relax the assumption that $N$ is equal across all contact networks. For detection, we followed the same method described in the main text with $N = 5$ or $N = 100$, $F = 200$, and $f_A = 0.5$ then compared the difference in power for these sample sizes. For parameter estimation and disease dynamics prediction, we followed the same method described in the main text for $N = 100$ with $C_d = C_c = 1.3$, $E_d = E_c = 0.25$, and $f_A = 0.2$. Since both $N$ and $F$ affect the sample size, we did this for $F = 200$ and $F = 5000$ to check if $N$ had a different effect with different $F$. To estimate parameters with $N = 5$, we used the same contact networks simulated for $N = 100$. This allowed us to compare differences due to $N$ while minimizing stochasticity from simulating the data. To do so, we randomly sampled the number of infected naive individuals $x_{i,s}$ in each network $i$ with $N = 5$ based on the fraction of naive individuals infected $\frac{x_{i,l}}{99}$ in $i$ with $N = 100$. Thus, $x_{i,s}$ has distribution $\text{Binom}(y = 4, p = \frac{x_{i,l}}{99})$. Since there is one focal individual in each network regardless of $N$, we used the same number of focal individuals infected (0 or 1) in $i$ for $N = 5$ as simulated for $N = 100$. After determining $x_{i,s}$ for all $i$, we ran MCMC and generated SIR dynamics as before.

We found that our power to detect heterogeneity in susceptibility and our ability to predict SIR dynamics were not substantially affected by increasing the number of individuals in each contact network, $N$, from 5 to 100 (Figs A, B, C, D, E). In certain parameter space, $N$ may have some importance for detection, but power is overall not that sensitive to $N$ (Figs A, B, C, D). We might have expected that higher $N$, or more naive individuals, would decrease variability in our estimate of $p_n$ and allow us to more precisely estimate parameters. However, there are $F(N - 1)$ naive individuals in the second exposure round, so even when $N$ is small there is not much variation in the fraction of naive individuals infected.

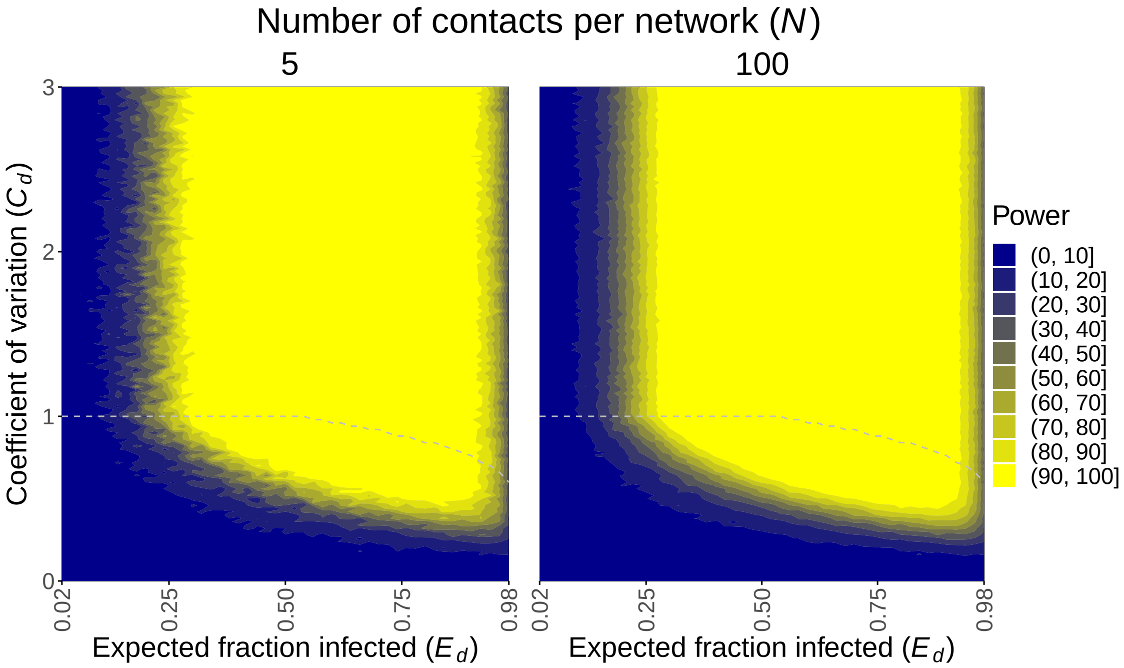


Figure A: Power to detect heterogeneity in susceptibility in the discrete case across different numbers of individuals in a contact network $N$. The areas above the gray dashed lines represent parameter space that gives computationally indistinguishable probabilities of infection $p_A$ and $p_B$, and therefore power, to the parameter combination with the same $E_d$ and highest $C_d$ below the line. This occurs because risks of infection can be changed to increase $C_d$ without bound, whereas probabilities are bounded at zero and one. $F = 200$ and $f_A = 0.5$.

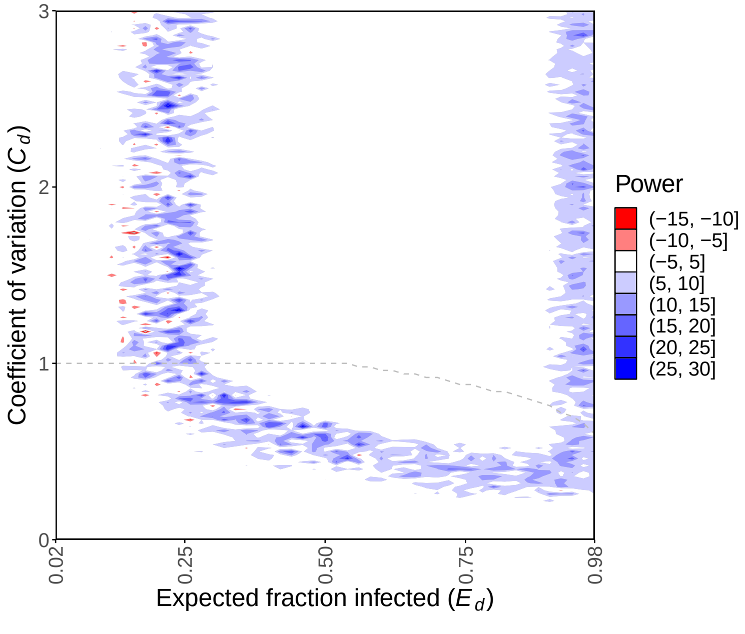


Figure B: There is generally more power to detect heterogeneity in susceptibility in the discrete case when $N = 100$ than when $N = 5$, but the effect is relatively small and restricted to the space where heterogeneity is sometimes detectable with either value of $N$. This plot shows the difference in the power to detect heterogeneity in susceptibility between $N = 5$ and $N = 100$ for the discrete case. Positive (blue) areas mean that there is more power to detect heterogeneity in susceptibility with $N = 100$, and negative (red) areas mean there is less power with $N = 100$. $F = 200$ and $f_A = 0.5$.

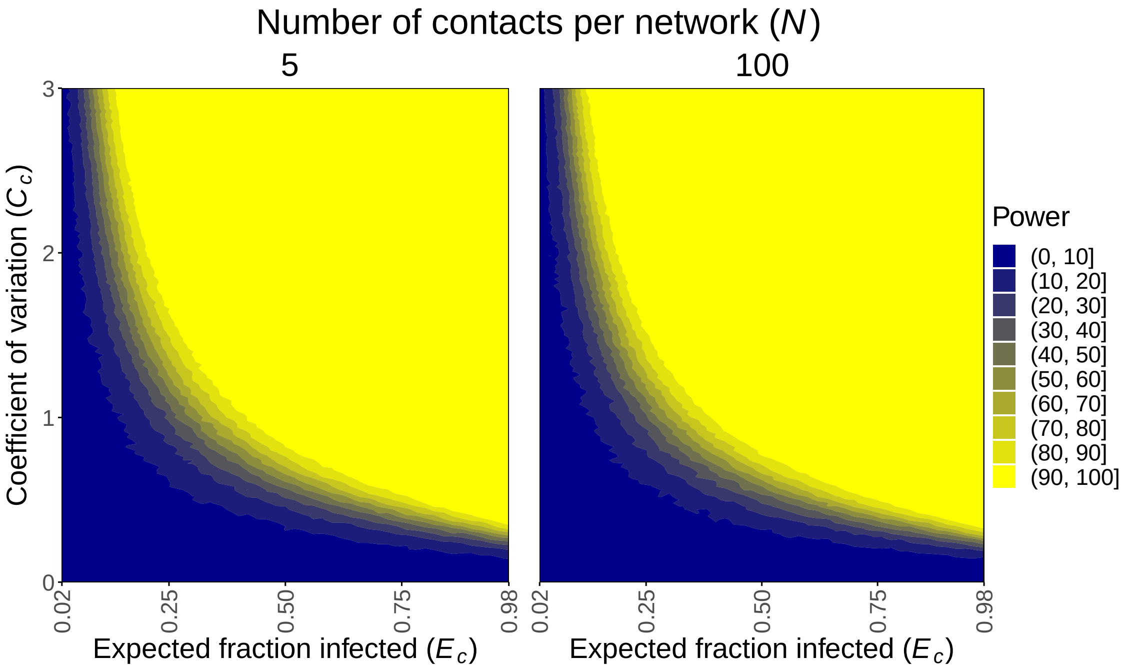


Figure C: Power to detect heterogeneity in susceptibility in the continuous case across different numbers of individuals in a contact network $N$. $F = 200$.

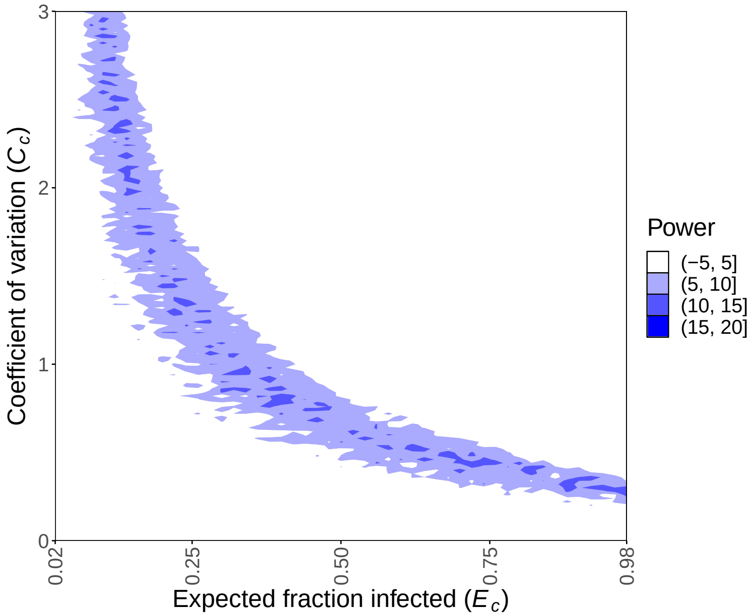


Figure D: There is generally more power to detect heterogeneity in susceptibility in the continuous case when $N = 100$ than when $N = 5$, but the effect is relatively small and restricted to the space where heterogeneity is sometimes detectable with either value of $N$. This plot shows the difference in the power to detect heterogeneity in susceptibility between $N = 5$ and $N = 100$ for the continuous case. Positive (blue) areas mean that there is more power to detect heterogeneity in susceptibility with $N = 100$. $F = 200$.

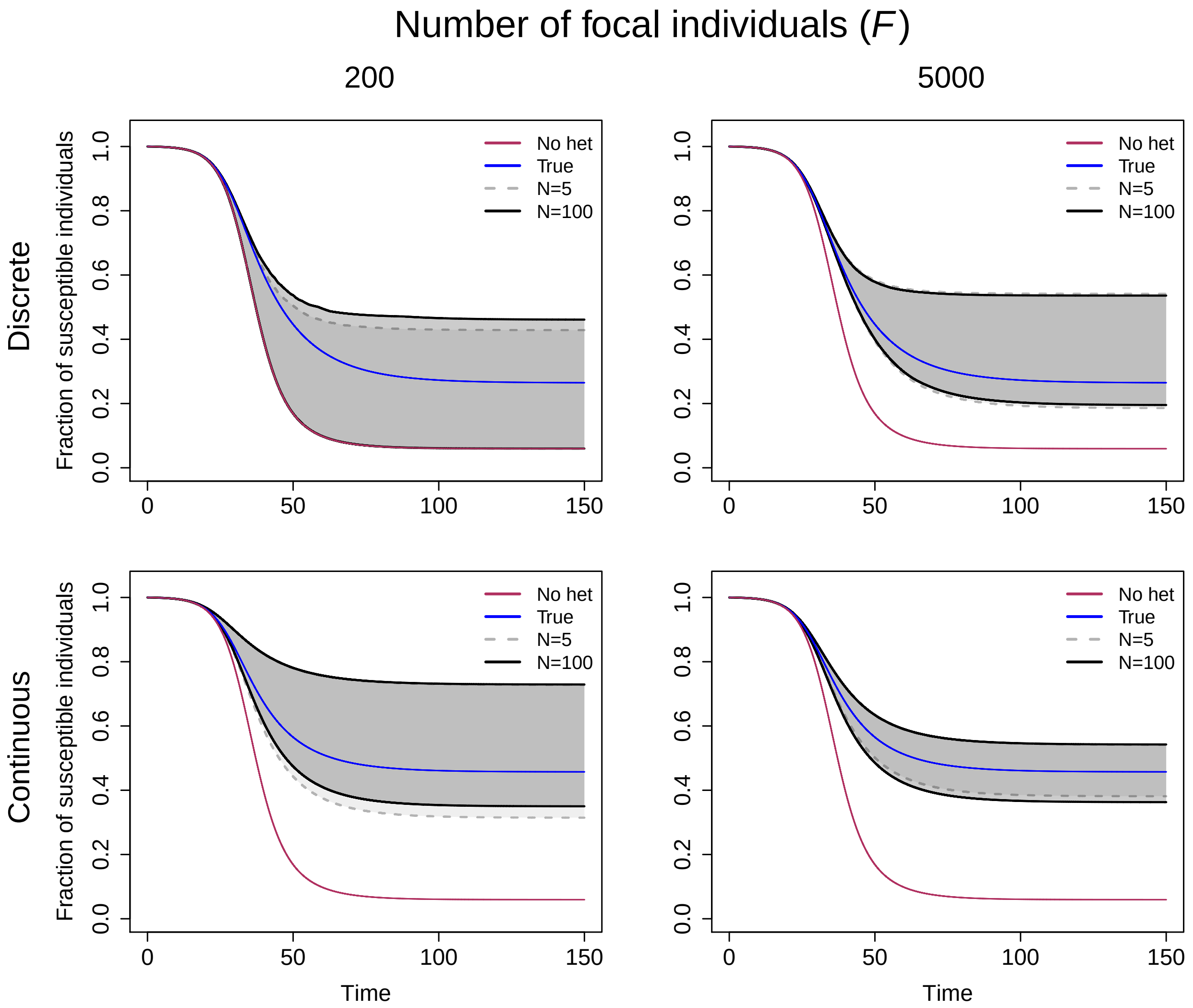


Figure E: The number of contacts $N$ does not substantially affect our prediction of the disease dynamics. The plots show the predicted SIR dynamics with $N = 5$ versus $N = 100$ contacts per network for both the discrete and the continuous case and different numbers of focal individuals $F$. Specifically, the fraction of susceptible individuals $\frac{S}{S_0}$ is shown over the course of an epidemic. Shaded regions represent 95% CIs determined from 1,000 posterior samples for $N = 5$ (gray) and $N = 100$ (black). The blue line shows the true dynamics for the parameters used to generate the contact tracing data, and the red line shows the corresponding dynamics if there is homogeneity in susceptibility. $C_d = C_c = 1.3$, $E_d = E_c = 0.25$, and $f_A = 0.2$.
