# Supplementary material for: Detecting and quantifying heterogeneity in susceptibility using contact tracing data
Source: PLoS Comput Biol. 2024 Jul 29;20(7):e1012310. doi: 10.1371/journal.pcbi.1012310 (PMC11309420; doi:10.1371/journal.pcbi.1012310)
Supplement: S3 Text — (PDF) [file pcbi.1012310.s003.pdf]

Supporting Information S3: Difference between probabilities of infection for naive and focal hosts (p_n and p_f) and variability in the likelihood ratio test statistic

Beth M. Tuschhoff, David A. Kennedy

Department of Biology, The Pennsylvania State University, University Park, Pennsylvania, United States of America

---

We found that our power to detect heterogeneity in susceptibility increased with larger sample sizes and greater heterogeneity present as well as an intermediate expected fraction of naive individuals infected E_d in the discrete case and a high expected fraction infected E_c in the continuous case. We wanted to further understand how these factors affect our power in terms of the likelihoods used to test for heterogeneity and the probabilities of infection for naive, focal, and average hosts p_n, p_f, and $\bar{p}$ in them. As p_n and $\bar{p}$ are typically very similar, we focused on investigating p_n and p_f. We theorized that the difference between p_n and p_f, $\Delta p = p_n - p_f$, may be important for detecting heterogeneity. To check this, we tested the hypothesis that p_n and p_f are different from each other against the null hypothesis that they are the same. We did this for $p_n \in [0, 1]$ with step size 0.01 and $p_f \in [0, p_f]$ with step size 0.01. Note that although we tested each combination of p_n and p_f to better understand and visualize our ability to detect heterogeneity, not all combinations are possible for data with coefficient of variation of risk $C \in [0, 3]$ and expected fraction infected $E \in [0.02, 0.98]$. In figure A, the area above the gray line is the parameter space that is possible in the discrete case with $F = 50$, $N = 5$, and $f_A = 0.5$.

For each parameter combination, we first set the probabilities of infection for naive and focal hosts p_n and p_f and simulated the number of naive and focal individuals infected with $F = 50$ and $N = 5$ where the number of naive individuals infected has distribution $\text{Binom}(y = F(N-1), p_n)$, and the number of focal individuals infected has distribution $\text{Binom}(y = F, p_f)$. We calculated the log-likelihood of the data the same as in the main text where L_{hom} is the log-likelihood under the null hypothesis that p_n and p_f are the same, and L_{het} is the log-likelihood under the alternative hypothesis that p_n and p_f are different and there is, therefore, heterogeneity in susceptibility. We then compared the log-likelihoods of the data under each hypothesis using a likelihood ratio test with one degree of freedom and significance level $\alpha = 0.05$. We ran $10,000$ simulations for each set of parameters to determine our statistical power to detect a difference between p_n and p_f or heterogeneity with that parameter combination, where power was defined as the percent of simulations in which a significant difference was detected.

Figure A shows that power increases as the difference in probability of infection Δp increases. But, the contour lines are curved such that there is less power as $p_n, p_f \to 0.5$ and more power as $p_n, p_f \to 0$ or $p_n, p_f \to 1$. We believe this pattern arises because there is stochasticity in which individuals become infected, and, with a binomial distribution, the highest variance is at a probability of 0.5. This stochasticity then leads to variability in the likelihood ratio test statistic, making it harder to detect a difference between p_n and p_f. Hence, the variance increases as $p_n, p_f \to 0.5$ and thus power decreases. Overall, we found that our ability to detect heterogeneity in susceptibility ultimately depends on Δp and variability in the likelihood ratio test statistic stemming from stochasticity in exposure events.

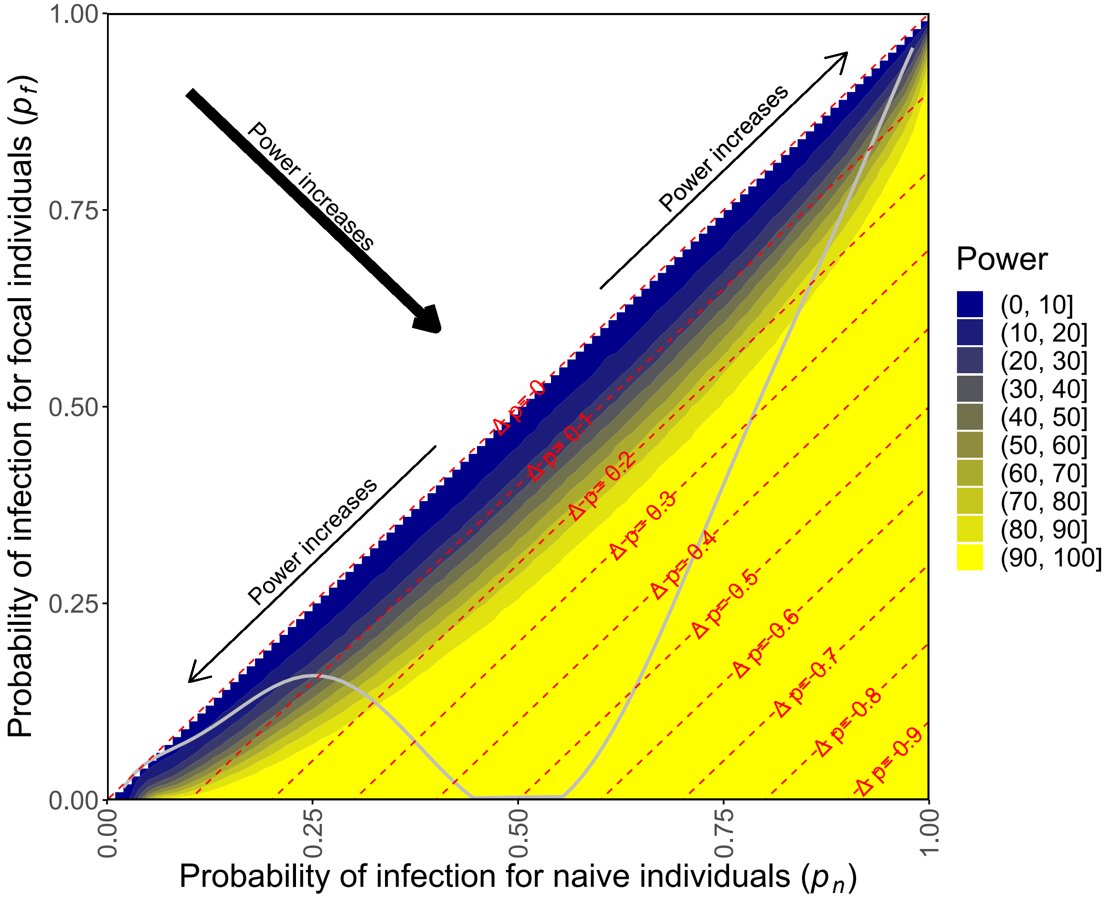

Figure A: The effects of $\Delta p = p_n - p_f$ and variability in the likelihood ratio test statistic on the power to detect heterogeneity in susceptibility. Power increases substantially as Δp increases, and power also increases slightly as variability in the test statistic decreases (i.e. as $p_n, p_f \to 0$ or $p_n, p_f \to 1$). The red dashed lines correspond to the values of Δp, and the arrows show how power increases with their thickness reflecting importance. The area above the gray line is the parameter space that is possible in the discrete case. All parameter space is possible in the continuous case given a large enough coefficient of variation C_c. $F = 50$, $f_A = 0.5$, and $N = 5$.
