# Supplementary material for: Detecting and quantifying heterogeneity in susceptibility using contact tracing data
Source: PLoS Comput Biol. 2024 Jul 29;20(7):e1012310. doi: 10.1371/journal.pcbi.1012310 (PMC11309420; doi:10.1371/journal.pcbi.1012310)
Supplement: S4 Text — (PDF) [file pcbi.1012310.s004.pdf]

Supporting Information S4: Changing error tolerance for Approximate Bayesian Computation (ABC) in the discrete case

Beth M. Tuschhoff, David A. Kennedy

Department of Biology, The Pennsylvania State University, University Park, Pennsylvania, United States of America

---

To estimate parameters using our method with data that follows a discrete distribution of susceptibilities, we used ABC [1]. This ABC method estimated the likelihood by simulating the data 100 times and examining the fraction of simulations that were close to our observed data. Notably, "close" can be defined in many ways. Here we explored the effect of our definition of close on the outcome of the model. To do so, we ran ABC where we estimated the likelihood from the fraction of simulations where the number of individuals infected was within a 10%, 1%, or 0% error tolerance of the number infected in the observed data with $F = 200$ or 1000 and $N = 5$. We then compared the resulting predicted SIR dynamics. We used two different values of F to check if the error tolerance allowed was more important for different sample sizes. We found that changing the error tolerance did not substantially impact the precision of the 95% CIs. This can be seen in Figure A by comparing the CIs for an error tolerance of 10% (light gray), 1% (dark gray), and 0% (black) in each panel.

References

1. Beaumont MA, Zhang W, Balding DJ. Approximate Bayesian computation in population genetics. Genetics. 2002;162(4):2025–2035.

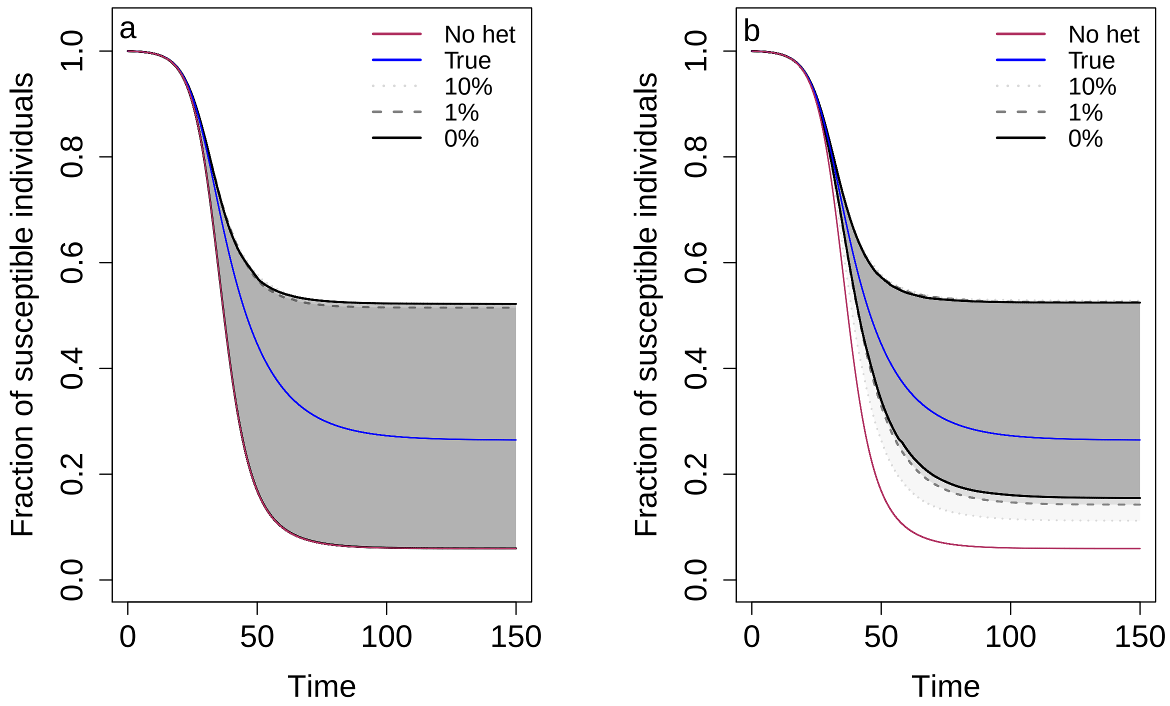

Figure A: The error tolerance allowed for ABC has little effect on our prediction of the disease dynamics. The plots show the predicted SIR dynamics in the discrete case with different error tolerances allowed for ABC with a) $F = 200$ and b) $F = 1000$. Specifically, the fraction of susceptible individuals $\frac{S}{S_0}$ is shown over the course of an epidemic. Shaded regions represent 95% CIs determined from 1,000 posterior samples for an error tolerance of 10% (light gray), 1% (dark gray), and 0% (black). The blue line shows the true dynamics for the parameters used to generate the contact tracing data, and the red line shows the corresponding dynamics if there is homogeneity in susceptibility. $C_d = 1.3$, $E_d = 0.25$, $f_A = 0.2$, and $N = 5$.
