# Supplementary material for: Detecting and quantifying heterogeneity in susceptibility using contact tracing data
Source: PLoS Comput Biol. 2024 Jul 29;20(7):e1012310. doi: 10.1371/journal.pcbi.1012310 (PMC11309420; doi:10.1371/journal.pcbi.1012310)
Supplement: S5 Text — (PDF) [file pcbi.1012310.s005.pdf]

Supporting Information S5: Effect of assuming the wrong underlying model

Beth M. Tuschhoff, David A. Kennedy

Department of Biology, The Pennsylvania State University, University Park, Pennsylvania, United States of America

When estimating parameters with our simulated data, we know whether individuals' risks follow a discrete or continuous distribution. However, in a real system, it may be unknown which underlying model is correct. We therefore explored the impact of assuming the wrong underlying model on our estimated parameters and predicted disease dynamics. To do so, we generated data under the discrete case then predicted SIR dynamics assuming the continuous case and vice versa. Figure A shows that the 95% CIs from the incorrectly assumed underlying models did not capture the true dynamics in either case. This is because the way individuals' risks are distributed in each underlying model results in fundamentally different epidemics. In the continuous case, there is a core group of highly resistant individuals that leads to a smaller epidemic size, whereas in the discrete case, when $f_A = 0.2$, the less susceptible type B individuals are not highly resistant ($p_B = 0.125$) and are therefore still reasonably likely to become infected leading to a larger epidemic relative to the continuous case. However, in the discrete case, when $f_A = 0.5$, the type B individuals are much more resistant ($p_B = 7.4e - 05$), so the 95% CIs from the incorrectly assumed continuous case better captured the true dynamics (Fig A). Overall, this demonstrates that the underlying model used should be carefully chosen to reflect prior understanding of the potential drivers of heterogeneity in susceptibility in the system. We note, however, that this complication only arises for parameter estimation and generating SIR dynamics. Our process for detecting heterogeneity in susceptibility does not depend on the underlying model, so we can reliably determine whether there is heterogeneity without knowledge of the distribution of individuals' risks.

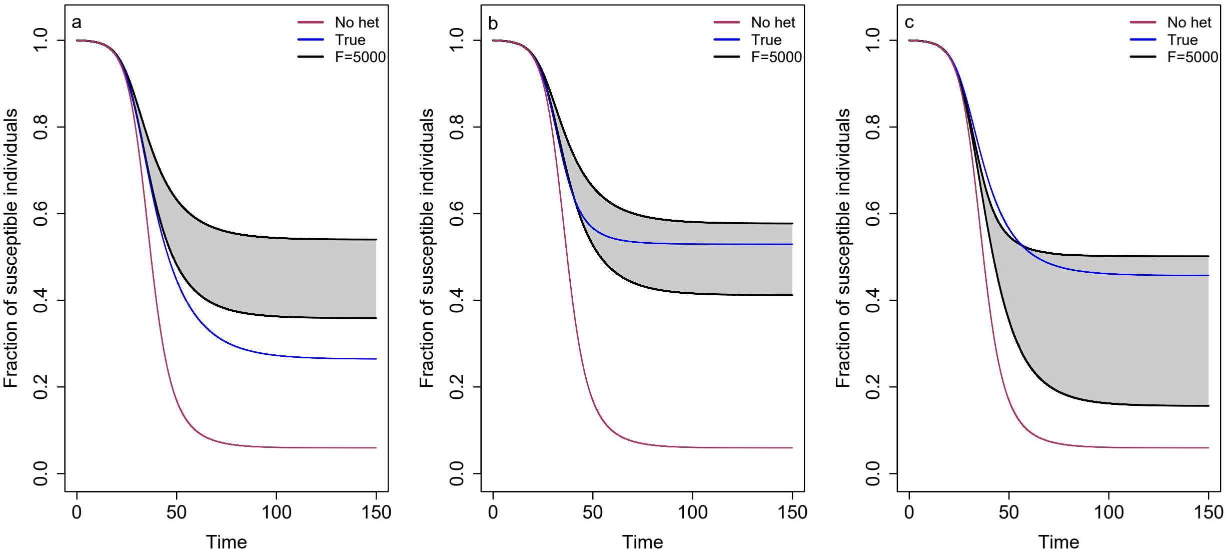

Figure A: Assuming the wrong underlying model for estimation results in incorrect predictions of the disease dynamics, but this effect is lessened as $f_A \to 0.5$ when the discrete case is the correct model. The plots show the predicted SIR dynamics with the wrong underlying model assumed for a) the discrete case with the continuous case incorrectly assumed and $f_A = 0.2$, b) the discrete case with the continuous case incorrectly assumed and $f_A = 0.5$, and c) the continuous case with the discrete case incorrectly assumed. Specifically, the fraction of susceptible individuals $\frac{S}{S_0}$ is shown over the course of an epidemic. Shaded regions represent 95% CIs determined from 1,000 posterior samples for $F = 5000$. The blue line shows the true dynamics for the parameters used to generate the contact tracing data, and the red line shows the corresponding dynamics if there is homogeneity in susceptibility. In each plot, the data was simulated according to the true underlying model, but parameters were estimated according to the wrong underlying model. $C_d = C_c = 1.3$, $E_d = E_c = 0.25$, and $N = 5$.
