# Supplementary material for: Detecting and quantifying heterogeneity in susceptibility using contact tracing data
Source: PLoS Comput Biol. 2024 Jul 29;20(7):e1012310. doi: 10.1371/journal.pcbi.1012310 (PMC11309420; doi:10.1371/journal.pcbi.1012310)
Supplement: S6 Text — (PDF) [file pcbi.1012310.s006.pdf]

Supporting Information S6: Final epidemic size in the discrete case with different fractions of the population that are more susceptible (f_A)

Beth M. Tuschhoff, David A. Kennedy

Department of Biology, The Pennsylvania State University, University Park, Pennsylvania, United States of America

---

When testing our method with different parameter combinations for the discrete case, we noticed that, with the same expected fraction infected E_d, we generated different powers to detect heterogeneity in susceptibility and different SIR dynamics by changing the fraction of individuals that was more susceptible f_A, even if the coefficient of variation of risk C_d was kept constant. To better understand the effect of f_A on disease dynamics in relation to C_d, we investigated the final epidemic size for different C_d and f_A. For each combination of $C_d \in [0, 3]$ and $f_A \in [0.05, 0.95]$ by step size 0.05, we computed p_A and p_B and ran the SIR dynamics as described in the main text. We set $E_d = 0.25$. We then calculated the final epidemic size as $1 - \frac{S_t}{S_0}$ where S_0 and S_t are the number of susceptible individuals at the beginning and end of the epidemic respectively.

We found that the final epidemic size depends on both C_d and f_A (Fig A). For a specific f_A, epidemic size decreases as C_d increases. This was expected as higher C_d, equivalently, more heterogeneity in susceptibility, is known to result in smaller epidemics [1]. However, for a specific C_d, there can be different epidemic sizes depending on f_A, and if both f_A and C_d are changed, it is possible to get a larger epidemic with a higher C_d. For a given $R_{0,d}$, the smallest epidemic is possible when $f_A = E_d$ and C_d is large. This is because, when $f_A = E_d$, $p_A \to 1$ and $p_B \to 0$ as C_d increases. So, with a large C_d, the largest possible final epidemic size here occurs when all the A individuals are infected. This gives an epidemic size of E_d. When f_A shifts away from E_d, p_A remains large but $p_B > 0$, so the epidemic can continue even if all A individuals are infected. This can then result in an epidemic size greater than E_d. Overall, in addition to C_d and E_d, the fraction of the population that is the more susceptible type of individual f_A is critical for determining the trajectory of the epidemic.

References

1. Ball F. Deterministic and stochastic epidemics with several kinds of susceptibles. Adv Appl Probab. 1985;17(1):1–22.

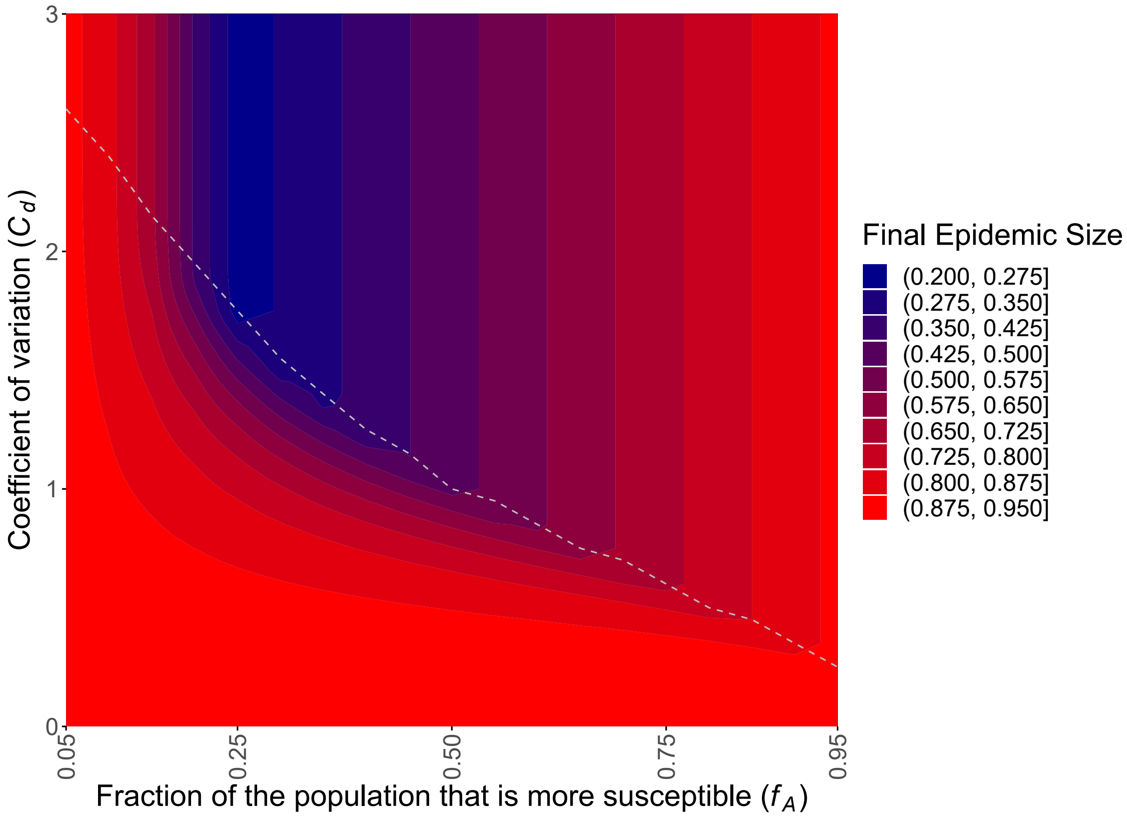

Figure A: Final epidemic size in the discrete case depends on both the coefficient of variation of risk C_d and fraction of the population that is more susceptible f_A. The plot shows the fraction of individuals infected over the course of an epidemic with varying C_d and f_A. The area above the gray dashed line represents parameter space that gives computationally indistinguishable probabilities of infection p_A and p_B, and therefore final epidemic size, to the parameter combination with the same f_A and highest C_d below the line. This occurs because risks of infection can be changed to increase C_d without bound, whereas probabilities are bounded between zero and one. Note that for a specific f_A, epidemic size decreases as C_d increases, but for a specific C_d, epidemic size differs depending on f_A. For a given $R_{0,d}$, the smallest epidemic is possible when $f_A = E_d$ and C_d is large. $E_d = 0.25$.
