# Supplementary material for: Detecting and quantifying heterogeneity in susceptibility using contact tracing data
Source: PLoS Comput Biol. 2024 Jul 29;20(7):e1012310. doi: 10.1371/journal.pcbi.1012310 (PMC11309420; doi:10.1371/journal.pcbi.1012310)
Supplement: S7 Text — (PDF) [file pcbi.1012310.s007.pdf]

Supporting Information S7: Effect of having informative priors for p_A, p_B, or f_A on predicted disease dynamics

Beth M. Tuschhoff, David A. Kennedy

Department of Biology, The Pennsylvania State University, University Park, Pennsylvania, United States of America

---

In the discrete case, we are unable to generate as precise of disease dynamics predictions as in the continuous case. This is because in the discrete case, we must estimate the three parameters p_A, p_B, and f_A to define the risk distribution, whereas in the continuous case, only two parameters k and θ must be estimated. In addition, the parameters are all highly correlated, leading to low identifiability. If, however, anything is previously known about the discrete parameters, then we may be able to more precisely estimate them by setting informative priors or by integrating other data sources. For example, if the factor creating heterogeneity in susceptibility is whether someone wears a mask or not, we might have an idea of f_A (i.e., the fraction of people that do not wear masks). To test this, we generated posterior distributions using Metropolis-Hastings MCMC and ABC as described in the main text but with informative priors instead of flat priors. For the priors, we used truncated normal distributions restricted to $[0, 1]$ with mean given by the true parameter value for p_A, p_B, or f_A and standard deviation 0.05. We then randomly sampled $1,000$ parameter sets from the posterior distribution to run SIR model simulations and determine 95% CIs. Unsurprisingly, we found that we could generate narrower 95% CIs with informative priors than with flat priors (Fig A). This indicates that prior knowledge of p_A, p_B, or f_A could improve the precision of disease dynamics predictions in the discrete case.

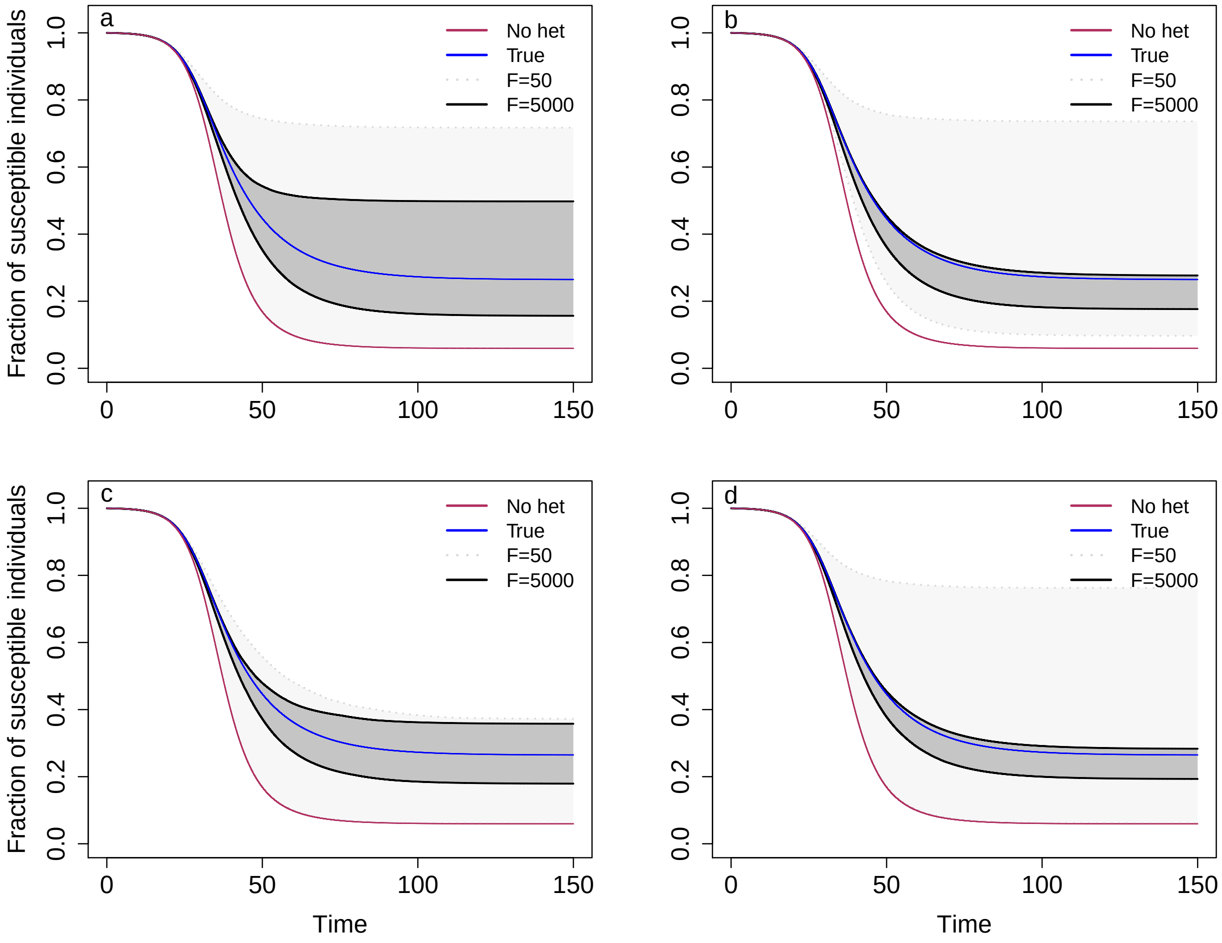

Figure A: Using informative priors for p_A, p_B, or f_A improves the precision of our predicted disease dynamics. The plots show the predicted SIR dynamics in the discrete case with a) uninformative priors for p_A, p_B, and f_A, b) an informative prior for p_A, c) an informative prior for p_B, and d) an informative prior for f_A. Specifically, the fraction of susceptible individuals $\frac{S}{S_0}$ is shown over the course of an epidemic. Shaded regions represent 95% CIs determined from 1,000 posterior samples for $F = 50$ (gray) or 5000 (black). The blue line shows the true dynamics for the parameters used to generate the contact tracing data, and the red line shows the corresponding dynamics if there is homogeneity in susceptibility. The informative priors used for p_A, p_B, and f_A were truncated normal distributions restricted to [0,1] with mean given by the true parameter value and standard deviation 0.05. $C_d = 1.3$, $E_d = 0.25$, $f_A = 0.2$, and $N = 5$.
