# Supplementary material for: Detecting and quantifying heterogeneity in susceptibility using contact tracing data
Source: PLoS Comput Biol. 2024 Jul 29;20(7):e1012310. doi: 10.1371/journal.pcbi.1012310 (PMC11309420; doi:10.1371/journal.pcbi.1012310)
Supplement: S8 Text — (PDF) [file pcbi.1012310.s008.pdf]

Supporting Information S8: False negatives

Beth M. Tuschhoff, David A. Kennedy

Department of Biology, The Pennsylvania State University, University Park, Pennsylvania, United States of America

---

While in the main text we have assumed access to perfect data, we can imagine that real contact tracing data may be imperfect. One way we have considered that data may be imperfect is that individuals may be mislabeled as uninfected when they are infected (false negatives). As we said in the main text, false negatives can cause our method to overestimate the level of heterogeneity in susceptibility because our estimate of the infection probability for focal individuals p_f may be biased lower. This is because, assuming infection confers at least partial immunity, focal individuals that were previously infected (i.e. false negatives) will be less likely to be infected than focal individuals that were true negatives. To counteract this issue, we developed a version of the method that corrects for false negatives by adjusting the likelihood calculations for both detecting and estimating heterogeneity in susceptibility. For detection, we first consider that x_I of the F focal individuals were false negatives and therefore incorrectly classified as focal with probability p_I, so we should only include $F - x_I$ focal individuals in the likelihood. Then, if we observe D_η naive individuals and D_F focal individuals infected in the data, there may be an additional x_η naive individuals and x_F focal individuals infected that we observed as uninfected where $x_\eta \in [0, F(N-1) - D_\eta]$ and $x_F \in [0, F - x_I - D_F]$. We changed the likelihood equations to the following in order to account for false negatives.

$$L_{\text{hom}} = \ln\left[\sum_{x_\eta=0}^{F(N-1)-D_\eta}\left[P(D_\eta + x_\eta|F(N-1), \bar{p}) * P(x_\eta|D_\eta + x_\eta, \epsilon)\right]\right] + \ln\left[\sum_{x_I=0}^{F}\sum_{x_F=0}^{F-x_I-D_F}\left[P(D_F + x_F|F - x_I, \bar{p}) * P(x_I|F, p_{I,\text{hom}}) * P(x_F|D_F + x_F, \epsilon)\right]\right] \quad (1)$$

$$L_{\text{het}} = \ln\left[\sum_{x_\eta=0}^{F(N-1)-D_\eta}\left[P(D_\eta + x_\eta|F(N-1), p_n) * P(x_\eta|D_\eta + x_\eta, \epsilon)\right]\right] + \ln\left[\sum_{x_I=0}^{F}\sum_{x_F=0}^{F-x_I-D_F}\left[P(D_F + x_F|F - x_I, p_f) * P(x_I|F, p_{I,\text{het}}) * P(x_F|D_F + x_F, \epsilon)\right]\right] \quad (2)$$

L_{hom} is the log-likelihood of the data under the null hypothesis that there is homogeneity in susceptibility, and L_{het} is the log-likelihood under the alternative hypothesis that there is heterogeneity in susceptibility. $P(x|y, p)$ is the probability of observing x individuals infected out of y individuals exposed with probability p of being infected and is distributed according to a binomial distribution. $D_\eta + x_\eta$ and $D_F + x_F$ are the numbers of naive and focal individuals actually infected respectively where D_η is the number of naive individuals observed infected in the data, D_F is the number of focal individuals observed infected in the data, and x_η and x_F are the numbers of naive and focal individuals infected but observed as uninfected (number of false negatives). $D_\eta + x_\eta \in [0, F(N-1)]$, $x_\eta \in [0, F(N-1) - D_\eta]$, $D_F + x_F \in [0, F - x_I]$, and $x_F \in [0, F - x_I - D_F]$. x_I is the number of individuals that were previously infected and incorrectly classified as focal because they were perceived to be uninfected. $x_I \in [0, F]$. ϵ is the false negative rate where $\epsilon \in [0, 1]$. $p_{I,\text{hom}}$ and $p_{I,\text{het}}$ are the probabilities that a focal individual was previously infected and incorrectly classified for the homogeneous and heterogeneous likelihoods respectively. $p_{I,\text{hom}} = \frac{\bar{p}\epsilon}{1-\bar{p}+\bar{p}\epsilon}$ and $p_{I,\text{het}} = \frac{p_n\epsilon}{1-p_n+p_n\epsilon}$. p_n, p_f and $\bar{p}$ are the true probabilities of infection and are calculated as $p_n = \frac{p_{n,\text{obs}}}{1-\epsilon}$, $p_f = \frac{p_{f,\text{obs}}F}{(F-x_I)(1-\epsilon)}$, and $\bar{p} = \frac{\bar{p}_{\text{obs}}FN}{(FN-x_I)(1-\epsilon)}$. $p_{n,\text{obs}}$, $p_{f,\text{obs}}$ and $\bar{p}_{\text{obs}}$ are the probabilities of infection observed from the data including false negatives and are estimated as $p_{n,\text{obs}} = \frac{D_\eta}{F(N-1)}$, $p_{f,\text{obs}} = \frac{D_F}{F}$, and $\bar{p}_{\text{obs}} = \frac{D_F+D_\eta}{FN}$.

For estimation, we also altered the likelihood calculations in our MCMC algorithm. In the discrete case, we implemented the same ABC framework as in the main text except we included false negatives at rate ϵ in our simulations. In the continuous case, we switched from an analytical likelihood to a simulated likelihood with ABC. We also increased our MCMC chain to length 1,200,000 with a burn-in of 500,000 and thinning interval 100. We followed the same procedure to calculate likelihood in the continuous case as in the discrete case.

For detecting heterogeneity in susceptibility, adjusting the likelihood calculation greatly lessened the impact of false negatives, but the method will still fail for values of E close to 1 (Figs A, B). This is because our likelihoods are approximations as we do not account for stochasticity in the observed number of infected individuals and the number of false negatives in our calculations. This means that the true probabilities of infection will be computed as greater than 1 if the observed probability of infection and false negative rate ϵ are large enough. For example, if $p_{n,\text{obs}} > 1 - \epsilon$, then $p_n > 1$. Since probabilities cannot exceed 1, we set an upper bound of 1 for the true probabilities. However, if there are no false negatives in the data, we could incorrectly compute a true probability of infection as 1 when it is actually less than 1. For values of E_{inf} close to 1, this can cause us to incorrectly detect or not detect heterogeneity in susceptibility. We do not think this will be a major issue though as E is typically less than 0.5 [1, 2, 3, 4, 5, 6]. For estimating parameters and predicting disease dynamics, adjusting the likelihoods to correct for false negatives fixed the issue. This is shown in Figure C where the 95% CI for the method adjusted to account for false negatives (black) captures the true disease dynamics, whereas the original, unadjusted method (gray) overestimates the level of heterogeneity, leading to the prediction of a smaller epidemic.

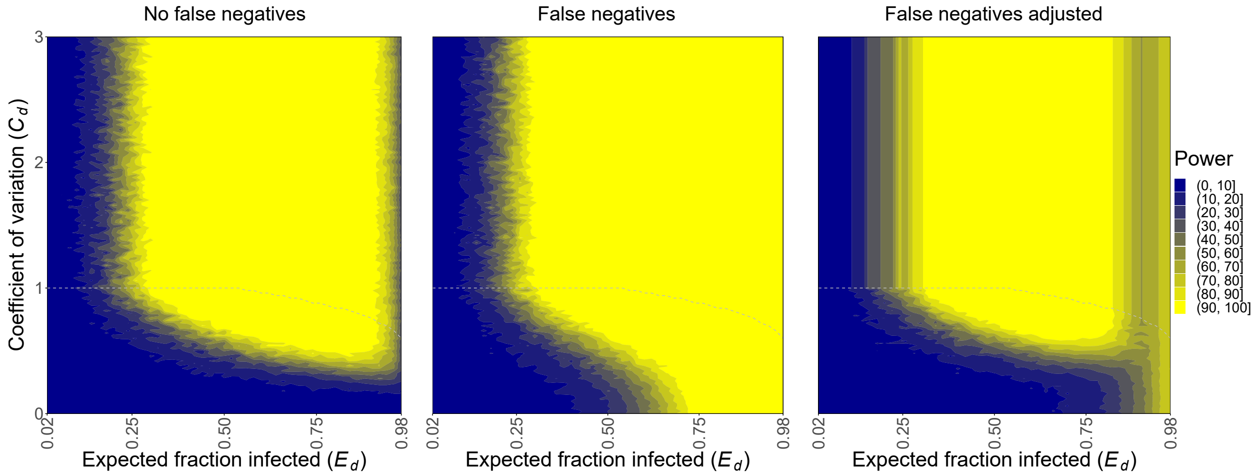

Figure A: The presence of false negatives in the discrete case causes our method to detect heterogeneity in susceptibility when we should not be able to, but adjusting the method corrects this issue except when E_d is close to 1. The plots show the power to detect heterogeneity in susceptibility in the discrete case with no false negatives, false negatives with the original method, and false negatives with the adjusted method. The areas above the gray dashed lines represent parameter space that gives computationally indistinguishable probabilities of infection p_A and p_B, and therefore power, to the parameter combination with the same E_d and highest C_d below the line. This occurs because risks of infection can be changed to increase C_d without bound, whereas probabilities are bounded. $F = 200$, $N = 5$, $f_A = 0.5$, and $\epsilon = 0.1$.

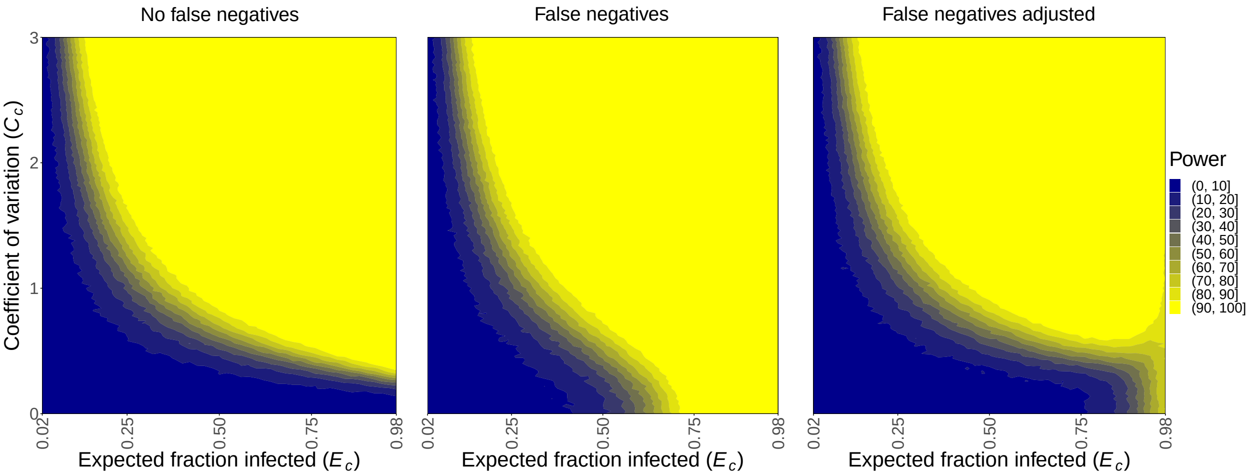

Figure B: The presence of false negatives in the continuous case causes our method to detect heterogeneity in susceptibility when we should not be able to, but adjusting the method corrects this issue except when E_c is close to 1. The plots show our power to detect heterogeneity in susceptibility in the continuous case with no false negatives, false negatives with the original method, and false negatives with the adjusted method. $F = 200$, $N = 5$, and $\epsilon = 0.1$.

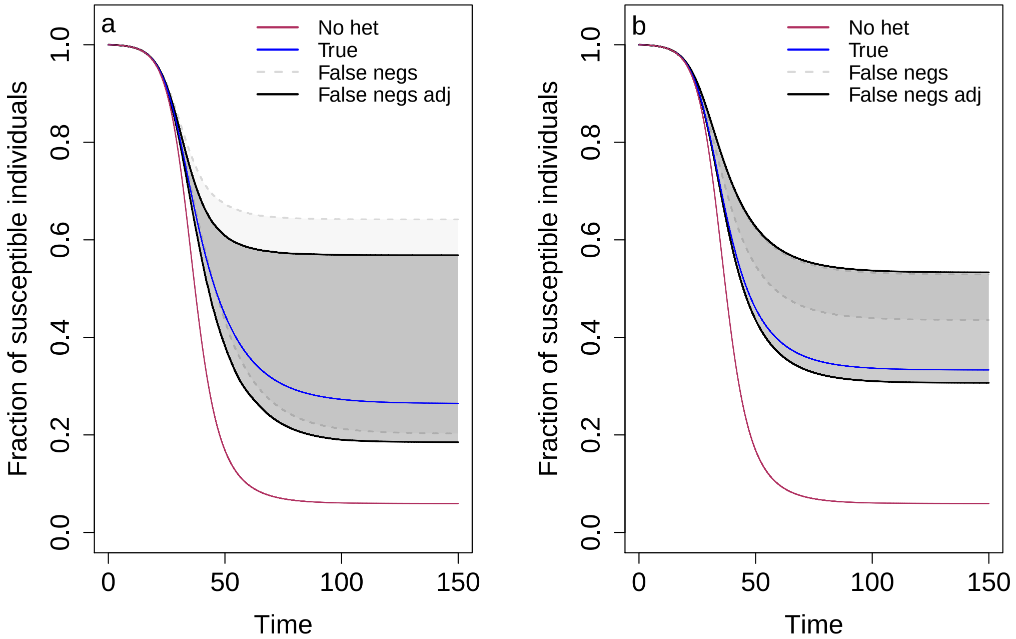

Figure C: The presence of false negatives causes our method to overestimate the level of heterogeneity in susceptibility, but adjusting the method corrects this issue. The plots show the predicted SIR dynamics in a) the discrete case and b) the continuous case with false negatives when we do or do not adjust the method to account for false negatives. Specifically, the fraction of susceptible individuals $\frac{S}{S_0}$ is shown over the course of an epidemic. Shaded regions represent 95% CIs determined from 1,000 posterior samples for the method adjusted to account for false negatives (black) and not adjusted to account for false negatives (gray). The blue line shows the true dynamics for the parameters used to generate the contact tracing data, and the red line shows the corresponding dynamics if there is homogeneity in susceptibility. $C_d = 1.3$, $E_d = 0.25$, $f_A = 0.2$, $\epsilon_d = 0.2$, $C_c = 1$, $E_c = 0.75$, $\epsilon_c = 0.1$, $F = 1000$, and $N = 5$.

References

1. Ajelli M, Parlamento S, Bome D, Kebbi A, Atzori A, Frasson C, et al. The 2014 Ebola virus disease outbreak in Pujehun, Sierra Leone: epidemiology and impact of interventions. BMC Med. 2015;13. 281.

2. Koh WC, Naing L, Chaw L, Rosledzana MA, Alikhan MF, Jamaludin SA, et al. What do we know about SARS-CoV-2 transmission? A systematic review and meta-analysis of the secondary attack rate and associated risk factors. PLoS One. 2020;15(10):e0240205.

3. Lessler J, Reich NG, Cummings DA, of Health NYCD, Team MHSII. Outbreak of 2009 pandemic influenza A (H1N1) at a New York City school. N Engl J Med. 2009;361:2628–2636.

4. Taylor MM, Rotblatt H, Brooks JT, Montoya J, Aynalem G, Smith L, et al. Epidemiologic investigation of a cluster of workplace HIV infections in the adult film industry: Los Angeles, California, 2004. Clin Infect Dis. 2007;44(2):301–305.

5. De Serres G, Shadmani R, Duval B, Boulianne N, Déry P, Fradet MD, et al. Morbidity of pertussis in adolescents and adults. J Infect Dis. 2000;182(1):174–179.

6. Rieder H. Contacts of tuberculosis patients in high-incidence countries. Int J Tuberc Lung Dis. 2003;7:S333–S336.
