# Supplementary material for: Detecting and quantifying heterogeneity in susceptibility using contact tracing data
Source: PLoS Comput Biol. 2024 Jul 29;20(7):e1012310. doi: 10.1371/journal.pcbi.1012310 (PMC11309420; doi:10.1371/journal.pcbi.1012310)
Supplement: S9 Text — (PDF) [file pcbi.1012310.s009.pdf]

Supporting Information S9: Heterogeneity in transmission

Beth M. Tuschhoff, David A. Kennedy

Department of Biology, The Pennsylvania State University, University Park, Pennsylvania, United States of America

---

As mentioned in the main text, heterogeneity in transmission, differences between individuals in their likelihood of infecting others, given contact may create problems for our method. This is because heterogeneity in transmission given contact may swamp out perceived differences in individuals' susceptibilities as force of infection in addition to innate susceptibility affect whether individuals become infected, so less susceptible individuals are not necessarily less likely to be infected. To test this, we attempted to detect and estimate heterogeneity in susceptibility given contact as well as predict SIR dynamics in the presence of heterogeneity in transmission given contact. We used the same methods as in the main text but simulated the data with heterogeneity in transmission in addition to heterogeneity in susceptibility. In order to add heterogeneity in transmission into our simulations, we generated a force of infection λ_i, $i = 1, ..., 2F$ to represent the infectiousness of the infected individual in each contact network where λ_i has distribution Gamma(m, ϕ), where $\phi = 1/m$ and the distribution thus has a mean of 1. The shape parameter m is a measure of the level of heterogeneity in transmission present where small m means that there is a lot of heterogeneity, and as $m \to \infty$, the level of heterogeneity decreases to zero. Here, we set $m = 0.5$ and $\phi = 2$ as this falls within the range of biologically reasonable shape parameters [1]. We chose the gamma distribution because it is flexible and is used as part of the Poisson-gamma mixture definition of the negative binomial distribution to describe the number of individuals infected by a particular infected individuals [1]. We then multiplied the force of infection by the risk of being infected given contact to compute the probability of infection for each individual as $p_{i,j} = 1 - e^{-\lambda_i r_j}$ for $j = A, B$ in the discrete case and $j = 1, ..., FN$ in the continuous case. We also calculated an average force of infection Λ across all networks to ensure that we kept the same average probability of infection at the beginning of the epidemic regardless of heterogeneity. This was also multiplied by the force of infection for each network and risk of being infected to give the probability of infection for each individual as $p_{i,j} = 1 - e^{-\lambda_i \Lambda r_j}$ for $j = A, B$ in the discrete case and $j = 1, ..., FN$ in the continuous case. We computed Λ by numerically solving for the Λ that made it so that the expected probability of infection without heterogeneity in transmission, $\mathrm{E}[1 - e^{-r_j}]$, equaled the expected probability of infection with heterogeneity in transmission, $\mathrm{E}[1 - e^{-\lambda_i \Lambda r_j}]$.

We found that our power to detect heterogeneity in susceptibility and our ability to predict SIR dynamics in the presence of heterogeneity in transmission were impacted considerably (Figs A, B, C). With heterogeneity in transmission given contact, our power was greatly reduced, and we made less precise predictions of the disease dynamics. We also predicted a larger final epidemic size in the continuous case as we underestimated the level of heterogeneity in susceptibility. This is because our method does not consider that the force of infection in each contact network affects whether individuals are infected in addition to their innate susceptibilities. Our method, in the form presented in the main text, is therefore not suitable for these cases. However, even though our power was reduced, we note that the presence of heterogeneity in transmission does not cause us to detect heterogeneity in susceptibility when it did not exist (i.e. $C = 0$). Additionally, the impact of heterogeneity in transmission would be smaller with a larger m (i.e. less heterogeneity in transmission) and non-existent if this heterogeneity was due to contact rate rather than innate differences. Heterogeneity in contact rate would simply mean that each network would have its own number of contacts N. Importantly, we note that this variation in contact rate is typically assumed to be the cause of heterogeneity in transmission [1] in which case our method would be suitable.

An initial step to manage the effect of heterogeneity in transmission given contact on our method is to test for its presence. This allows us to determine whether the assumption of our base model that there is no heterogeneity in transmission given contact is violated. If there is not heterogeneity in transmission given contact, the number of naive individuals infected in each contact network will be binomially distributed

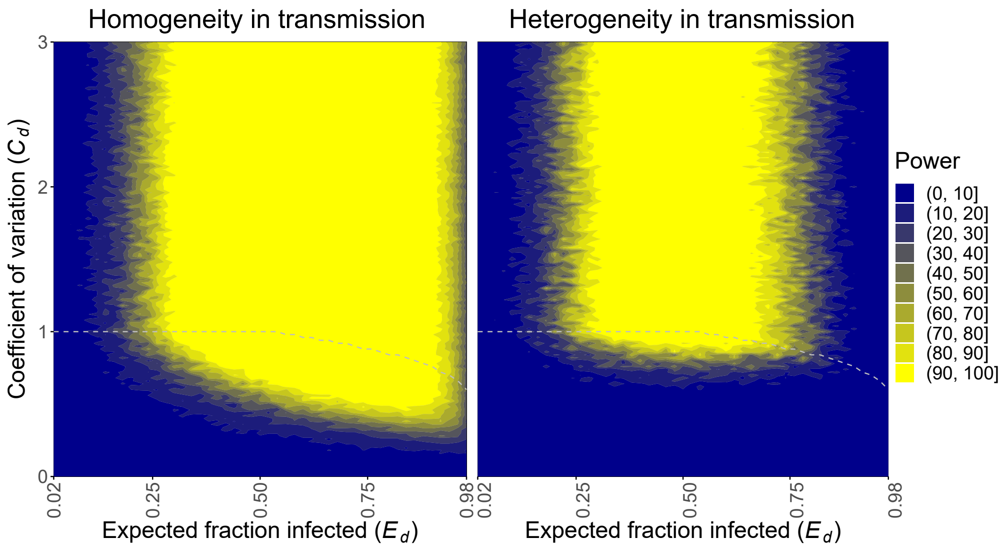

Figure A: The presence of heterogeneity in transmission given contact reduces our power to detect heterogeneity in susceptibility in the discrete case. The plots show the power to detect heterogeneity in susceptibility in the discrete case with homogeneity in transmission and with heterogeneity in transmission. The areas above the gray dashed lines represent parameter space that gives computationally indistinguishable probabilities of infection p_A and p_B, and therefore power, to the parameter combination with the same E_d and highest C_d below the line. This occurs because risks of infection can be changed to increase C_d without bound, whereas probabilities are bounded. $F = 200$, $N = 5$, $f_A = 0.5$, $m = 0.5$, and $\phi = 2$.

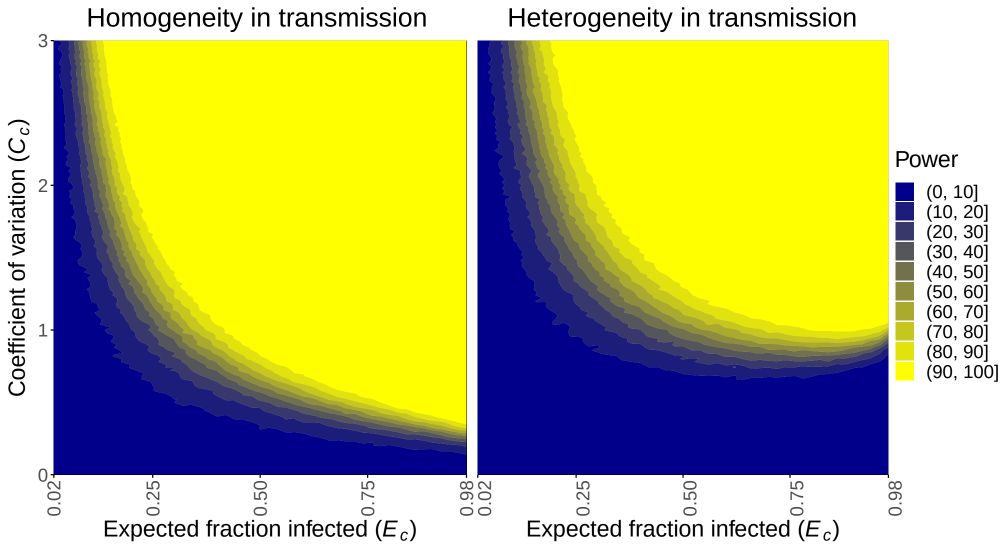

Figure B: The presence of heterogeneity in transmission given contact reduces our power to detect heterogeneity in susceptibility in the continuous case. The plots show the power to detect heterogeneity in susceptibility in the continuous case with homogeneity in transmission and with heterogeneity in transmission. $F = 200$, $N = 5$, $m = 0.5$, and $\phi = 2$.

regardless of the presence of heterogeneity in susceptibility given contact. This is because individuals are as likely to be infected or not if they are in contact with different hosts. And, when there is heterogeneity in susceptibility, individuals with higher and lower susceptibilities are binomially distributed across networks. If there is heterogeneity in transmission, we would expect to see a disproportionate number of contact networks where most or few individuals are infected. In the extreme case, either everyone or no one will be infected in each network. This is because the force of infection in each network will strongly affect whether individuals in that network are infected.

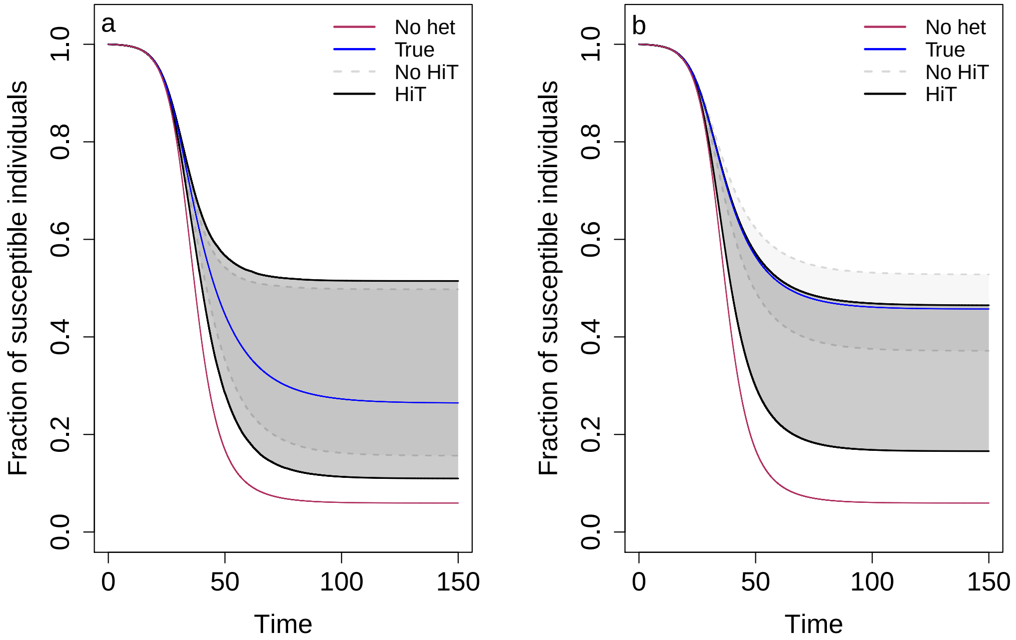

Figure C: The presence of heterogeneity in transmission given contact decreases the precision of our prediction of the disease dynamics and can cause our method to underestimate the level of heterogeneity in susceptibility. The plots show the effect of heterogeneity in transmission (HiT) on predicted SIR dynamics in a) the discrete case and b) the continuous case. Specifically, the fraction of susceptible individuals $\frac{S}{S_0}$ is shown over the course of an epidemic. Shaded regions represent 95% CIs determined from 1,000 posterior samples for homogeneity in transmission (gray) and heterogeneity in transmission (black). The blue line shows the true dynamics for the parameters used to generate the contact tracing data, and the red line shows the corresponding dynamics if there is homogeneity in susceptibility. $C_d = C_c = 1.3$, $E_d = E_c = 0.25$, $f_A = 0.2$, $F = 5000$, $N = 5$, $m = 0.5$, and $\phi = 2$.

To test for heterogeneity in transmission given contact, we can perform a goodness of fit test that compares the distribution of infection in naive individuals among contact networks to see if it follows a single binomial distribution. We use only naive individuals so that there is no effect on average susceptibility from focal individuals in case there is heterogeneity in susceptibility. To do so, we essentially simulate the contact networks many times with a binomial distribution and compare the likelihoods of those data to the real data to check if the real data are consistent with homogeneity in transmission. We first calculate an overall probability of infection p_n across all networks as the number of naive individuals infected divided by the number of naive individuals exposed. Next, we simulate the number of naive individuals infected in each network $10,000$ times according to a binomial distribution with probability p_n of infection. We can then calculate the log-likelihood of the real data as well as the log-likelihood of each set of simulated data as $\sum_{i=1}^{F} \ln[P(x_i|N_i, p_n)]$. x_i is the number of naive individuals infected in network i and N_i is the number of naive individuals exposed in network i. If the log-likelihood of the real data is within the 95% CI of the simulated log-likelihoods, we conclude that there is not significant heterogeneity in transmission given contact. If not, we conclude that there is heterogeneity in transmission given contact, and, therefore, our method, in the form presented in the main text, to detect heterogeneity in susceptibility would be underpowered, and estimates of heterogeneity in susceptibility would be unreliable (Fig D).

We tested the ability of this method to detect heterogeneity in transmission for parameters of the distribution dictating the level of heterogeneity in transmission $m \in [0.1, 6]$ with step size 0.1 and $\phi = \frac{1}{m}$. We examined $m \in [0.1, 6]$ because this captures a range of published estimates for this parameter: 0.16 to 5.1 [1]. We also set $C_d = C_c = 1.3$, $E_d = E_c = 0.25$, $f_A = 0.2$, $N = 5$, and $F = 200$ or 1000. This was done for $1,000$ simulations to compute the statistical power of the method. Our power here is the percent of simulations in which the log-likelihood of the real data lies outside the 95% CI of the simulated log-likelihoods (i.e. we

detect heterogeneity in transmission given contact).

Figure E shows that the sample size and level of heterogeneity affect our power to detect heterogeneity in transmission. As the number of focal individuals F increases from 200 to 1000 and as the number of contacts per network N increases from 5 to 10, there is greater power to detect all levels of heterogeneity in transmission (m). This was to be expected because a higher sample size improves the precision of our likelihoods. The level of heterogeneity in transmission present is described by the shape parameter m of the distribution of forces of infection. As m decreases, there is more power to detect heterogeneity in transmission as there is more heterogeneity in the population.

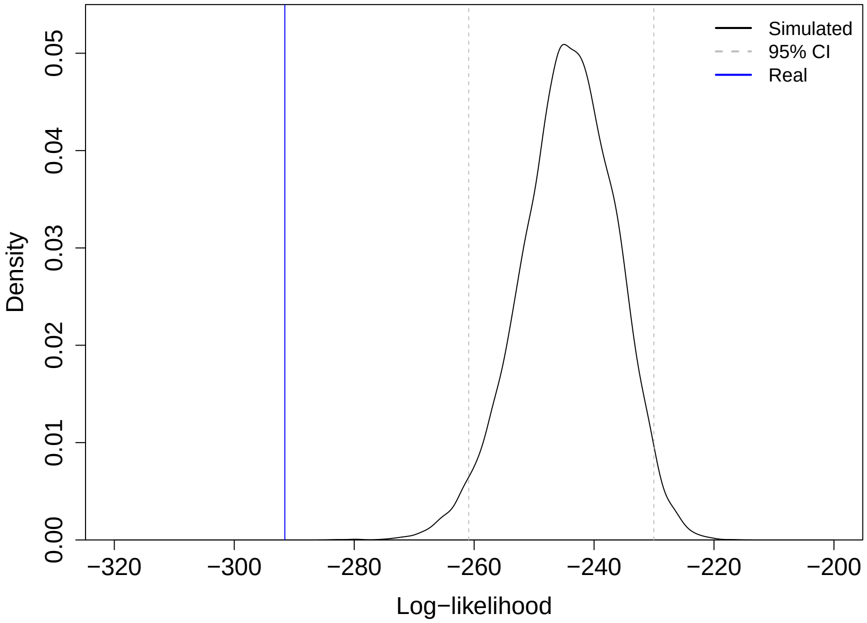

Figure D: Example of the method to detect heterogeneity in transmission given contact. The black line shows the distribution of simulated log-likelihoods, the gray dashed lines show the 95% CI of the simulated log-likelihoods, and the blue line shows the log-likelihood of the real data. Here, we would conclude that there is heterogeneity in transmission as the log-likelihood of the real data falls outside the 95% CI of the simulated log-likelihoods. $C_d = 1.3$, $E_d = 0.25$, $f_A = 0.2$, $F = 200$, $N = 5$, $m = 0.5$, and $\phi = 2$.

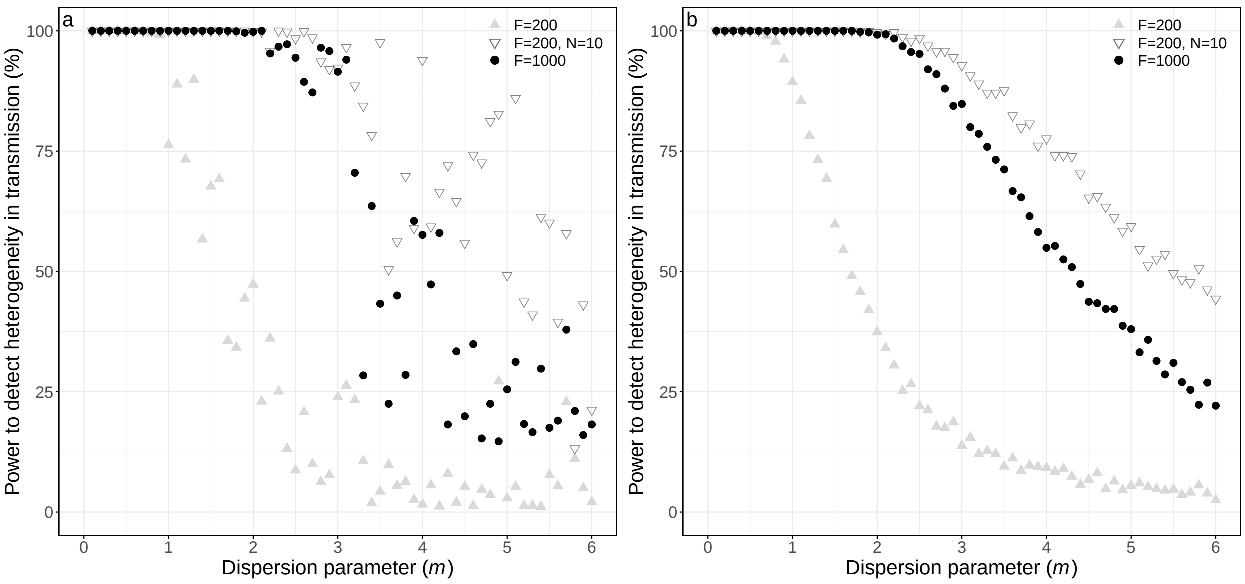

Figure E: An increased sample size (larger F and N) and increased level of heterogeneity in transmission given contact (smaller m) enhance our power to detect heterogeneity in transmission given contact. The plots show the power to detect heterogeneity in transmission given contact in the a) discrete case and b) continuous case in the presence of heterogeneity in susceptibility across varying levels of heterogeneity in transmission. Specifically, they show the percent of simulations in which the distribution of infection in naive individuals among contact networks is not binomial, so we conclude that there is heterogeneity in transmission given contact for $F = 200$ and $N = 5$ (light gray triangles), $F = 200$ and $N = 10$ (dark gray triangles), or $F = 1000$ and $N = 5$ (black circles). Heterogeneity in transmission is gamma distributed with shape parameter m and scale parameter $\phi = \frac{1}{m}$. $C_d = C_c = 1.3$, $E_d = E_c = 0.25$, and $f_A = 0.2$.

References

1. Lloyd-Smith JO, Schreiber SJ, Kopp PE, Getz WM. Superspreading and the effect of individual variation on disease emergence. Nature. 2005;438:355–359.
