# Supplementary material for: Detecting and quantifying heterogeneity in susceptibility using contact tracing data
Source: PLoS Comput Biol. 2024 Jul 29;20(7):e1012310. doi: 10.1371/journal.pcbi.1012310 (PMC11309420; doi:10.1371/journal.pcbi.1012310)
Supplement: S10 Text — (PDF) [file pcbi.1012310.s010.pdf]

Supporting Information S10: Estimating heterogeneity in susceptibility in the presence of heterogeneity in transmission given contact

Beth M. Tuschhoff, David A. Kennedy

Department of Biology, The Pennsylvania State University, University Park, Pennsylvania, United States of America

---

From this initial step, if we detect heterogeneity in transmission given contact, as stated above, our base method should not be used. Instead, we developed a generalized version of the method to account for both heterogeneity in susceptibility and heterogeneity in transmission by estimating parameters dictating the levels of both types of heterogeneity in the population and adjusting the likelihood calculations in our MCMC algorithm. We fit the parameters of our underlying risk distribution (k, θ) and underlying distribution of forces of infection (m, $\phi = \frac{1}{m}$) where the average force of infection Λ is encapsulated in our estimate of θ. So, we are estimating three parameters in total.

In our likelihood calculation, we now consider infection in the first exposure events in addition to the second as including the first round allows us to better disentangle the level of each type of heterogeneity in the population. For example, if there is a high force of infection in a first exposure event, we would expect any uninfected focal individuals coming from this event to be on average the most resistant individuals, resulting in a larger difference in infection probability between naive and focal individuals. In contrast, if there is a low force of infection in a first exposure event, some focal individuals may still be highly susceptible, reducing the difference in infection probability between naive and focal individuals. To calculate likelihood, for each set of parameters proposed, we simulate both exposure events $6,000$ times ($F = 6000$) and determine the number $W_{n1,n2,f}$ of these simulations in which x_{n1} naive individuals are infected in the first exposure event, x_{n2} naive individuals are infected in the second exposure event, and x_f focal individuals are infected in the second exposure event where $x_{n1} \in [0, N-1]$, $x_{n2} \in [0, N-1]$, and $x_f \in \{0, 1\}$. We then estimate the probability of each outcome $\{x_{n1}, x_{n2}, x_f\}$ as $p_{n1,n2,f} = \frac{W_{n1,n2,f}+1}{F+2}$. The likelihood of the data is then computed under a multinomial distribution with probability $p_{n1,n2,f}$ for each event $\{x_{n1}, x_{n2}, x_f\}$.

Our MCMC chains had differing lengths with a burn-in of 10,000 and thinning interval 200. We used exponential priors Exp(1) for k and Exp(2) for m because known values of C_c and m suggest that k and m are likely to be small [1, 2, 3, 4, 5, 6, 7, 8, 9, 10, 11, 12, 13]. We used an exponential prior Exp(0.2) for θ because we expect θ to be relatively small. We proposed k and θ separately from m. We used the same multivariate lognormal proposal distribution as in the main text for k and θ: $(k, \theta) \sim \text{MLogNorm}(\mu = \binom{0}{0}, \Sigma = \begin{pmatrix} 0.01 & -0.008 \\ -0.008 & 0.05 \end{pmatrix})$. We used a lognormal proposal distribution for m: $m \sim \text{LogNorm}(\mu = 0, \sigma = 0.2)$. We assessed convergence of the chains by visually inspecting the resulting trace plots and marginal posterior distributions for each parameter [14].

To investigate our ability to estimate parameters when both types of heterogeneity are present, we tested several parameter combinations. Here, we show our results for a high level of heterogeneity in susceptibility and high level of heterogeneity in transmission ($C_c = 1.3$, $E_c = 0.25$, $m = 0.5$) and a low level of heterogeneity in susceptibility and low level of heterogeneity in transmission ($C_c = 0.8$, $E_c = 0.75$, $m = 1$). We also tested our method with a low level of heterogeneity in susceptibility and high level of heterogeneity in transmission ($C_c = 0.8$, $E_c = 0.75$, $m = 0.5$), but for this scenario, our MCMC chain had extremely poor mixing with our proposal distribution and thus failed to converge. Nevertheless, in this latter case we do know that there is heterogeneity in transmission given contact from the goodness of fit test (S9 Text).

We found that when there is a high level of heterogeneity in susceptibility and/or a low level of heterogeneity in transmission, we are able to accurately estimate both heterogeneity in susceptibility and heterogeneity in transmission (Figs A, B). We determined our 95% CIs for parameter estimation with $F = 1000$ and $N = 5$ to be those shown in Table A. The true values for k, θ, and m as well as the composite parameter C_c are captured by these intervals. The true value for E_c is also captured in the low heterogeneity case but not in the high heterogeneity case. This may seem like a problem for predicting disease dynamics, but it

is not. Assuming that heterogeneity in susceptibility and heterogeneity in transmission are not correlated within individuals, the SIR dynamics depend only on C_c out of our estimated parameters. This is because heterogeneity in transmission only affects the probability of an epidemic taking off and its explosiveness [4]. In this case, we can use the same SIR model as in the main text for prediction, and in that model, we only use $\rho = E_c$ to calculate the contact rate c from an assumed $R_{0,c}$. Therefore, we can still accurately predict disease dynamics. While we have demonstrated a way to modify the method to account for both heterogeneity in susceptibility and transmission in the continuous case, this same type of modification could be applied in the discrete case.

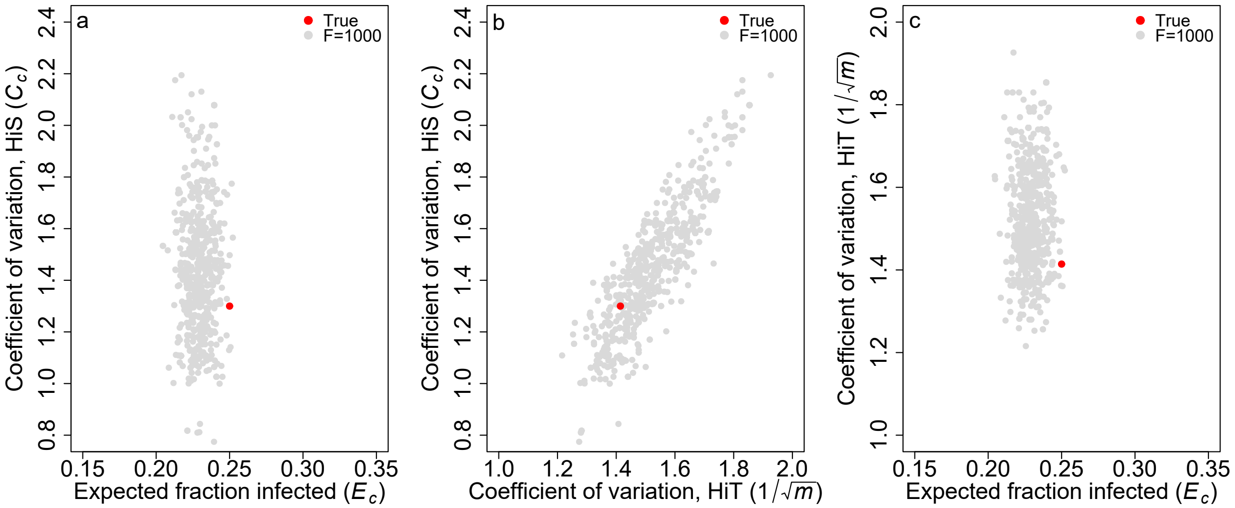

Figure A: Parameter estimates for the coefficient of variation for heterogeneity in susceptibility (C_c), expected fraction of naive individuals infected (E_c), and coefficient of variation for heterogeneity in transmission ($1/\sqrt{m}$) capture the true values for C_c and $1/\sqrt{m}$ in the case with high heterogeneity in susceptibility (HiS) and high heterogeneity in transmission (HiT). The plots show the parameter estimates for a) C_c vs. E_c, b) C_C vs $1/\sqrt{m}$, and c) $1/\sqrt{m}$ vs. E_c. The red dot represents the true parameters used to generate our simulated data, and the gray dots depict 1,000 parameter sets from our posterior distribution. $C_c = 1.3$, $E_c = 0.25$, $m = 0.5$, $1/\sqrt{m} = 1.414$, $F = 1000$, and $N = 5$.

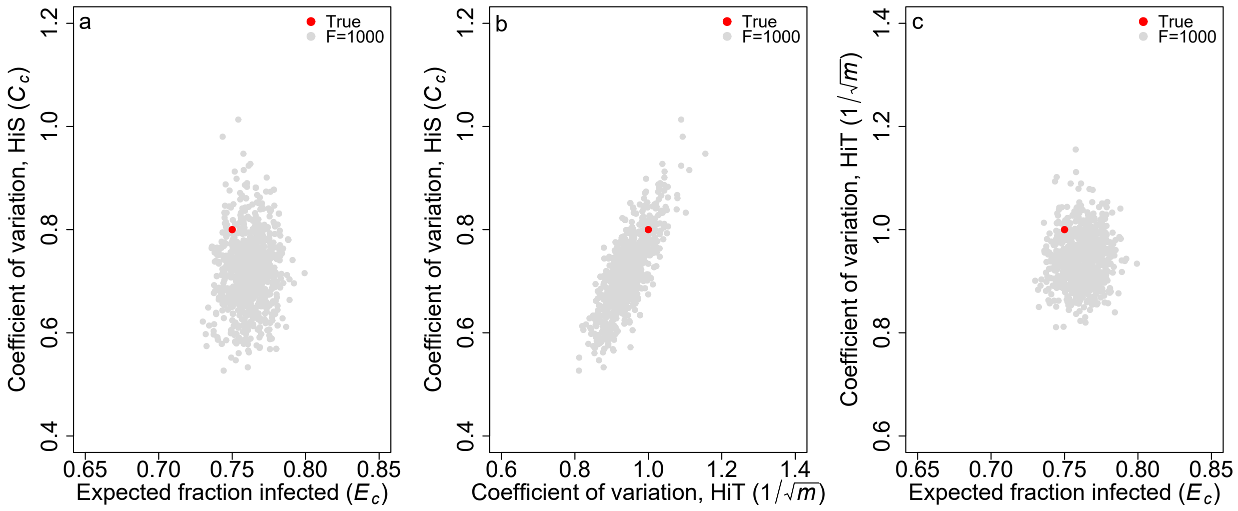

Figure B: Parameter estimates for the coefficient of variation for heterogeneity in susceptibility (C_c), expected fraction of naive individuals infected (E_c), and coefficient of variation for heterogeneity in transmission ($1/\sqrt{m}$) capture the true values in the case with low heterogeneity in susceptibility (HiS) and low heterogeneity in transmission (HiT). The plots show the parameter estimates for a) C_c vs. E_c, b) C_C vs $1/\sqrt{m}$, and c) $1/\sqrt{m}$ vs. E_c. The red dot represents the true parameters used to generate our simulated data, and the gray dots depict 1,000 parameter sets from our posterior distribution. $C_c = 0.8$, $E_c = 0.75$, $m = 1$, $1/\sqrt{m} = 1$, $F = 1000$, and $N = 5$.

Table A: The 95% CIs, medians, and true values for parameters estimated from MCMC for different levels of heterogeneity in susceptibility (HiS) and heterogeneity in transmission (HiT) with $F = 1000$ and $N = 5$. Note that the average force of infection Λ is encapsulated in our estimate of θ for MCMC, so we show this as a single parameter.

	Parameter	95% CI	Median	True
High HiS, High HiT	k	[0.262,0.883]	0.511	0.592
	$\theta\Lambda$	[0.522,5.028]	1.189	1.089
	m	[0.314,0.559]	0.454	0.5
	C_c	[1.064,1.954]	1.399	1.3
	E_c	[0.214,0.244]	0.229	0.25
Low HiS, Low HiT	k	[1.333,2.904]	1.930	1.563
	$\theta\Lambda$	[1.150,4.706]	2.305	3.231
	m	[0.937,1.354]	1.123	1
	C_c	[0.587,0.866]	0.720	0.8
	E_c	[0.740,0.782]	0.762	0.75

References

1. Dwyer G, Elkinton JS, Buonaccorsi JP. Host heterogeneity in susceptibility and disease dynamics: tests of a mathematical model. Am Nat. 1997;150(6):685–707.
2. Dwyer G, Dushoff J, Elkinton JS, Levin SA. Pathogen-driven outbreaks in forest defoliators revisited: building models from experimental data. Am Nat. 2000;156(2):105–120.
3. Smith D, Dushoff J, Snow R, Hay S. The entomological inoculation rate and Plasmodium falciparum infection in African children. Nature. 2005;438:492–495.
4. Lloyd-Smith JO, Schreiber SJ, Kopp PE, Getz WM. Superspreading and the effect of individual variation on disease emergence. Nature. 2005;438:355–359.
5. Ben-Ami F, Regoes RR, Ebert D. A quantitative test of the relationship between parasite dose and infection probability across different host–parasite combinations. Proc R Soc B. 2008;275:853–859.
6. Elderd BD, Dushoff J, Dwyer G. Host-pathogen interactions, insect outbreaks, and natural selection for disease resistance. Am Nat. 2008;172(6):829–842.
7. Ben-Ami F, Ebert D, Regoes RR. Pathogen dose infectivity curves as a method to analyze the distribution of host susceptibility: a quantitative assessment of maternal effects after food stress and pathogen exposure. Am Nat. 2010;175(1):106–115.
8. Pessoa D, Souto-Maior C, Gjini E, Lopes JS, Ceña B, Codeço CT, et al. Unveiling time in dose-response models to infer host susceptibility to pathogens. PLoS Comput Biol. 2014;10(8):e1003773.
9. Langwig KE, Wargo AR, Jones DR, Viss JR, Rutan BJ, Egan NA, et al. Vaccine effects on heterogeneity in susceptibility and implications for population health management. mBio. 2017;8:e00796–17.
10. King JG, Souto-Maior C, Sartori LM, Maciel-de Freitas R, Gomes MGM. Variation in Wolbachia effects on Aedes mosquitoes as a determinant of invasiveness and vectorial capacity. Nat Commun. 2018;9. 1483.
11. Gomes MGM, Oliveira JF, Bertolde A, Ayabina D, Nguyen TA, Maciel EL, et al. Introducing risk inequality metrics in tuberculosis policy development. Nat Commun. 2019;10. 2480.
12. Corder RM, Ferreira MU, Gomes MGM. Modelling the epidemiology of residual Plasmodium vivax malaria in a heterogeneous host population: A case study in the Amazon Basin. PLoS Comput Biol. 2020;16(3):e1007377.
13. Gomes MGM, Ferreira MU, Corder RM, King JG, Souto-Maior C, Penha-Gonçalves C, et al. Individual variation in susceptibility or exposure to SARS-CoV-2 lowers the herd immunity threshold. J Theor Biol. 2022;540. 111063.
14. Kennedy DA, Dukic V, Dwyer G. Combining principal component analysis with parameter line-searches to improve the efficacy of Metropolis–Hastings MCMC. Environ Ecol Stat. 2015;22:247–274.
