# Supplementary material for: Detecting and quantifying heterogeneity in susceptibility using contact tracing data
Source: PLoS Comput Biol. 2024 Jul 29;20(7):e1012310. doi: 10.1371/journal.pcbi.1012310 (PMC11309420; doi:10.1371/journal.pcbi.1012310)
Supplement: S11 Text — (PDF) [file pcbi.1012310.s011.pdf]

Supporting Information S11: Contact tracing data from a dynamic epidemic

Beth M. Tuschhoff, David A. Kennedy

Department of Biology, The Pennsylvania State University, University Park, Pennsylvania, United States of America

In our simulation of contact tracing data in the main text, we set up static contact networks, all of the same size N, and simulated infection within each network. To generate more realistic contact tracing data from a dynamic epidemic, we implemented a stochastic, individual-based SIR model for both the discrete and continuous case. In this model, we classified individuals as susceptible and naive (S_n), susceptible and previously exposed but not infected (i.e., focal; S_f), infected (I), or recovered (R). We assumed all susceptible individuals start as naive and move into the S_f class once they are exposed to an infected individual without being infected. At the start of each simulation, we set parameters dictating the level of heterogeneity present in the population (C_d, E_d, f_A or C_c, E_c), the basic reproduction number ($R_{0,d}$ or $R_{0,c}$), the recovery rate (γ), the average exposure or contact rate ($\bar{c}$), and the number of naive susceptible individuals and infected individuals at the start of the epidemic ($S_{n,0}$ and I_0). We calculated $\bar{c}$ from $R_{0,d}$ or $R_{0,c}$ where $R_{0,d} = \frac{(p_A f_A + p_B(1-f_A))c(S_{n,0}+I_0)}{\gamma}$ and $R_{0,c} = \frac{\rho\bar{c}(S_{n,0}+I_0)}{\gamma}$. $\rho = 1-(1+\theta)^{-k}$ is the average probability of being infected in the continuous case. We then assigned each individual i a risk of being infected r_i from the risk distribution dictated by p_A, p_B, and f_A in the discrete case or $r_i \sim \text{Gamma}(k, \theta)$ in the continuous case. p_A and p_B are calculated from C_d, E_d, and f_A, and k and θ are calculated from C_c and E_c. We randomly selected I_0 individuals to start as infected.

We then simulated the epidemic using a Gillespie algorithm. At each time point, an infected individual either exposes and potentially infects a susceptible individual or an infected individual recovers. Exposure occurs at rate $\bar{c}\sum_{i=1}^{I}\sum_{j=1}^{S_n+S_f} c_{i,j}$ where $c_{i,j}$ is the rate at which individuals i and j come in contact. We kept $c_{i,j}$ constant at 1, giving an exposure rate of $\bar{c}I(S_n+S_f)$ across all I infected and S_n+S_f susceptible individuals. If exposure occurs, we select an infected individual and a susceptible individual as the exposed individual. Then, we check if the susceptible individual is infected with infection probability $p_i = 1-e^{-r_i}$ for the ith individual. If the individual is infected, they move to the I class. If the individual is not infected and is naive, they are now previously exposed and move from S_n to the S_f class. If the individual is not infected and is already previously exposed, they remain in the S_f class. We record the number of naive individuals exposed, naive individuals infected, focal individuals exposed, and focal individuals infected by each I individual as that individual's contact network. Recovery occurs at rate γI across all I infected individuals. If recovery occurs, we randomly select an infected individual to recover. Upon recovery, the contact network of that individual is output as our contact tracing data.

Once we collected all of the data, we removed all infected individuals who did not expose anyone and all contact networks that did not contain at least one focal and one naive individual. To compare the results from these data with our results from the main text, we determined the first set of contact networks where at least F focal individuals were exposed and used these networks as our datasets. We then checked whether we could detect heterogeneity in susceptibility, estimate the parameters dictating the underlying risk distribution, and predict the associated SIR dynamics for these datasets, using the same methods described in the main text.

We tested the ability of our method to detect heterogeneity in susceptibility for $F = 50$ and $f_A = 0.5$ for each potential combination of $C_d \in [0, 3]$ with step size 0.1 and $E_d \in [0.14, 0.86]$ with step size 0.04 in the discrete case and $C_c \in [0, 3]$ with step size 0.1 and $E_c \in [0.14, 0.86]$ with step size 0.04 in the continuous case. This was done for 100 simulations to compute the statistical power of the method. We tested a sparser grid with fewer simulations here compared to the main text to alleviate computational effort. We also restricted E_d and E_c to be within 0.14 and 0.86 to more fairly compare detection power here with power from static networks in the main text as datasets generated from values outside these bounds tend to not include a set of networks with close to $F = 50$ focal individuals exposed. When E is small, many individuals are exposed

multiple times without being infected, so $F >> 50$. When E is large, many individuals are infected upon first exposure, so $F << 50$. For detection simulations, we set $R_{0,d} = R_{0,c} = 3$, $\gamma = 0.1$, $S_{n,0} = 499$, and $I_0 = 1$, giving a population size of $S_{n,0} + I_0 = 500$.

We also tested the ability of our method to estimate parameters dictating the level of heterogeneity in susceptibility and predict the associated SIR dynamics for $F = 50$, 200, 1000, or 5000. For these simulations, as in the main text, we set $C_d = C_c = 1.3$, $E_d = E_c = 0.25$, and $f_A = 0.2$ from which we calculated $p_A = 0.748$ and $p_B = 0.125$ or $k = 0.592$ and $\theta = 0.626$ that dictate the distribution of individuals' risks in the discrete or continuous case respectively. We also set $R_{0,d} = R_{0,c} = 3$, $\gamma = 0.1$, $S_{n,0} = 9,990$, and $I_0 = 10$, giving a population size of $S_{n,0} + I_0 = 10,000$. We followed the same MCMC protocol as in the main text, except our chains had differing lengths, and we used a burn-in of 100,000 in the continuous case. We also switched from an uninformative to an exponential prior Exp(0.2) for θ with $F = 50$ in the continuous case as the MCMC chain had trouble converging with this small of a sample size otherwise, and we expect θ to be relatively small.

We found that our method yields the same results for detection whether the contact networks are generated statically or dynamically. For estimation, our method yields the same results except in the case when focal individuals have been previously exposed many times without being accounted for. In both the discrete and continuous case, the power to detect heterogeneity in susceptibility is not substantially different for the different modes of simulating contact networks (Figs A, B). Note that because we used a coarse grid and 100 simulations per parameter set for the dynamically generated networks, resolution is lower than in the main text. There is also not a substantial difference in our accuracy and precision in predicting disease dynamics in the discrete case (Fig C). Note that we would not expect the 95% CIs to be the same here as they were calculated from different datasets. However, the general trends and conclusions drawn from the 95% CIs are the same for the two ways of simulating contact networks.

In the continuous case, our method is as precise with the dynamically generated contact networks, but it is not accurate for $F = 5000$ (Fig D). With $F = 50$, 200 and 1000, we are as precise and accurate in predicting disease dynamics with contact networks generated dynamically as we are with contact networks generated statically. Note that we would not expect the 95% CIs to be the same here as they were calculated from different datasets. With $F = 5000$, we do not accurately predict disease dynamics with the dynamically generated contact networks, but this is not as concerning as it seems at first. Here, our method is picking up on an artifact of how we set up our simulation of the dynamic epidemic. In the simulation, we grouped all previously exposed individuals in the focal category, regardless of their number of previous exposures. However, individuals that were exposed twice will on average be less susceptible than individuals that were exposed once, and the same would be true for individuals exposed three times. The number of exposures, however, is not currently recorded in our dataset. Later in the epidemic when many individuals will have been exposed many times, this biases our estimate of p_f lower, causing us to overestimate the level of heterogeneity in the population. This is because we are including a larger proportion of the most highly resistant individuals in the focal category as the epidemic progresses and not accounting for them.

By using a dynamic model for data generation, we have therefore shown that our method can be reliably applied to contact tracing data from a dynamic epidemic early in the epidemic and is not impacted by variable contact network sizes (variable N). Later in the epidemic, if focal individuals are previously exposed many times and this number of exposures is not accounted for, the method will lead to biased estimates and should therefore be modified.

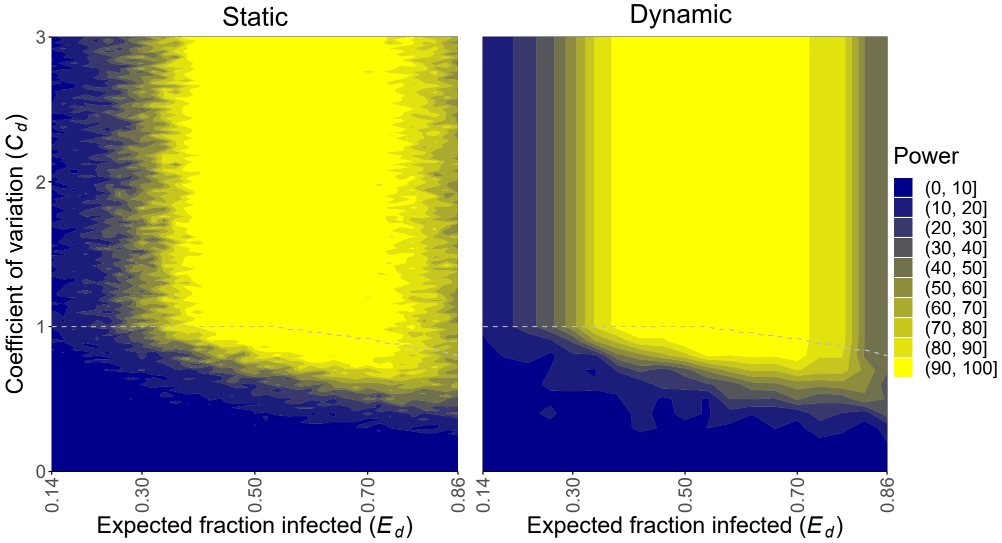

Figure A: Power to detect heterogeneity in susceptibility is not different when contact networks are generated statically versus dynamically in the discrete case. The plots show the power to detect heterogeneity in susceptibility in the discrete case where the contact networks are generated statically or from a dynamic epidemic. The areas above the gray dashed lines represent parameter space that gives computationally indistinguishable probabilities of infection p_A and p_B, and therefore power, to the parameter combination with the same E_d and highest C_d below the line. This occurs because risks of infection can be changed to increase C_d without bound, whereas probabilities are bounded. The power is calculated as described in the main text from 1,000 simulations with step size 0.02 in the static case and 100 simulations with step size 0.04 in the dynamic case. $F = 50$, $f_A = 0.5$, $N = 5$ in the static case, and N is variable in the dynamic case.

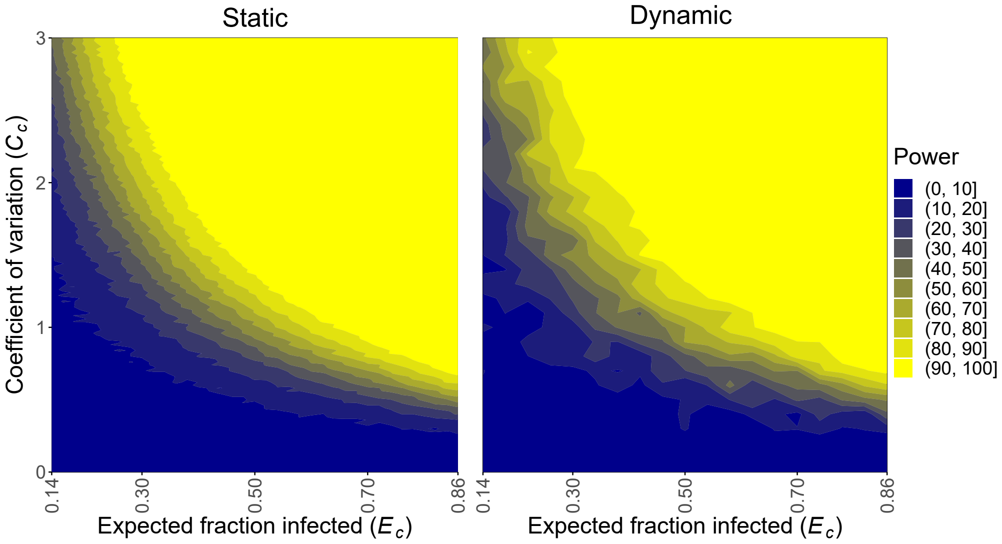

Figure B: Power to detect heterogeneity in susceptibility is not different when contact networks are generated statically versus dynamically in the continuous case. The plots show the power to detect heterogeneity in susceptibility in the continuous case where the contact networks are generated statically or from a dynamic epidemic. The power is calculated as described in the main text from 1,000 simulations with step size 0.02 in the static case and 100 simulations with step size 0.04 in the dynamic case. $F = 50$, $N = 5$ in the static case, and N is variable in the dynamic case.

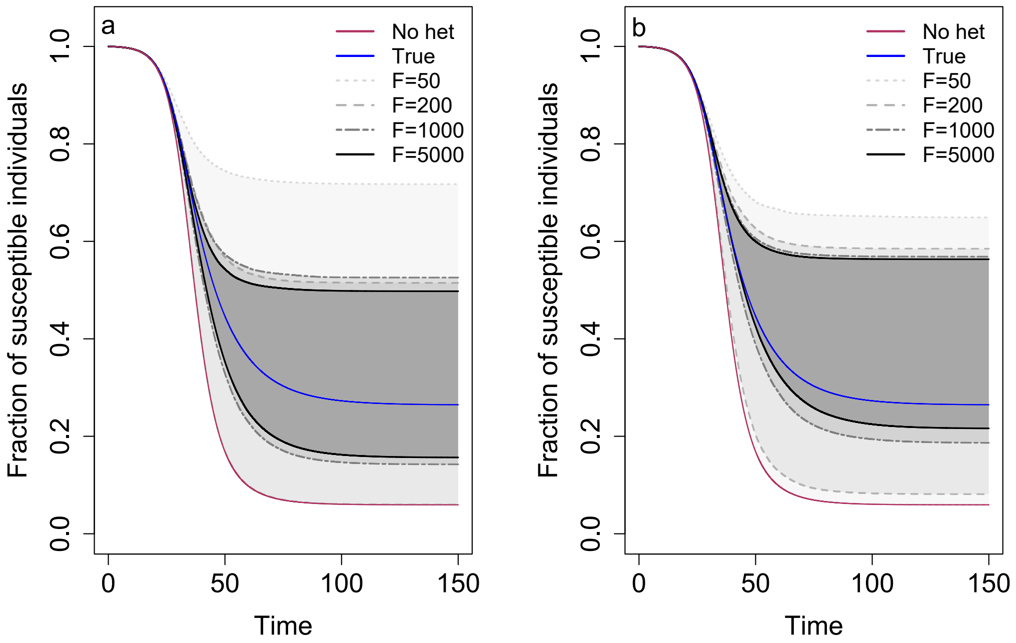

Figure C: Predicting disease dynamics based on contact networks that came from a dynamic epidemic does not substantially affect the accuracy and precision of our method in the discrete case. The plots show the predicted SIR dynamics in the discrete case where the contact networks are generated a) statically and b) dynamically with different numbers of focal individuals F. Specifically, the fraction of susceptible individuals $\frac{S}{S_0}$ is shown over the course of an epidemic. Shaded regions represent 95% CIs determined from 1,000 posterior samples for $F = 50$ (light gray), 200 (medium gray), 1000 (dark gray), and 5000 (black). For the disease dynamics predicted from contact networks generated dynamically (b), $F = 50$, 201, 1002, and 5000. The blue line shows the true dynamics for the parameters used to generate the contact tracing data, and the red line shows the corresponding dynamics if there is homogeneity in susceptibility. Note that differences in the parameter estimates between panels a and b are expected because of randomness in the data. $C_d = 1.3$, $E_d = 0.25$, $f_A = 0.2$, $N = 5$ in the static case, and N is variable in the dynamic case.

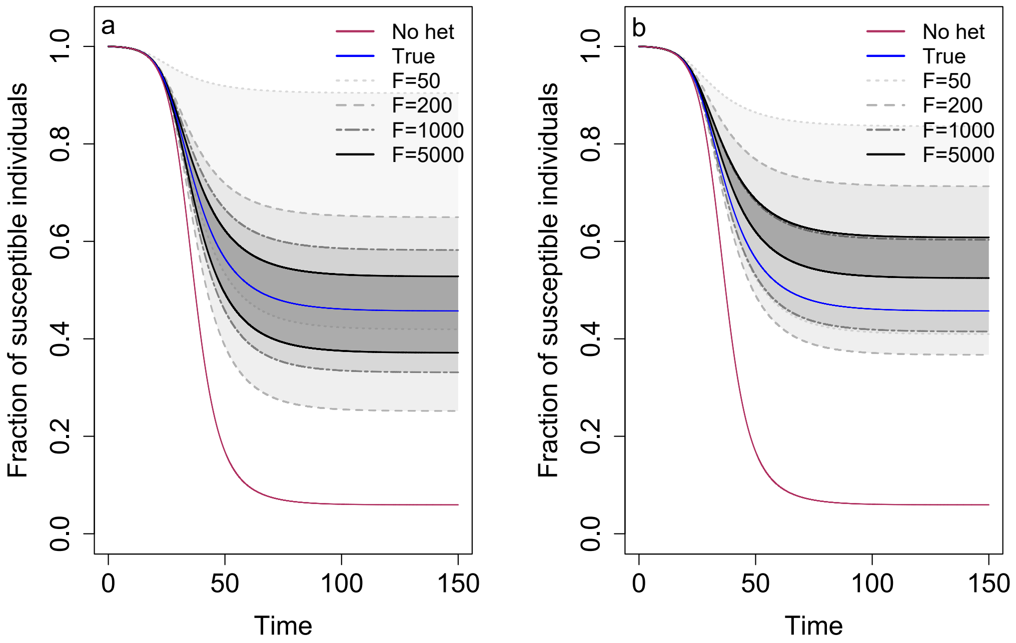

Figure D: Predicting disease dynamics based on contact networks that came from a dynamic epidemic does not substantially affect the precision of our method in the continuous case but reduces the accuracy when $F = 5000$. The plots show the predicted SIR dynamics in the continuous case where the contact networks are generated a) statically and b) dynamically with different numbers of focal individuals F. Specifically, the fraction of susceptible individuals $\frac{S}{S_0}$ is shown over the course of an epidemic. Shaded regions represent 95% CIs determined from 1,000 posterior samples for $F = 50$ (light gray), 200 (medium gray), 1000 (dark gray), and 5000 (black). For the disease dynamics predicted from contact networks generated dynamically (b), $F = 51$, 201, 1002, and 5005. The blue line shows the true dynamics for the parameters used to generate the contact tracing data, and the red line shows the corresponding dynamics if there is homogeneity in susceptibility. Note that differences in the parameter estimates between panels a and b are expected because of randomness in the data. $C_d = 1.3$, $E_d = 0.25$, $f_A = 0.2$, $N = 5$ in the static case, and N is variable in the dynamic case.
