# Supplementary material for: Detecting and quantifying heterogeneity in susceptibility using contact tracing data
Source: PLoS Comput Biol. 2024 Jul 29;20(7):e1012310. doi: 10.1371/journal.pcbi.1012310 (PMC11309420; doi:10.1371/journal.pcbi.1012310)
Supplement: S12 Text — (PDF) [file pcbi.1012310.s012.pdf]

Supporting Information S12: Missing contacts

Beth M. Tuschhoff, David A. Kennedy

Department of Biology, The Pennsylvania State University, University Park, Pennsylvania, United States of America

---

In the main text, we assumed that all contacts were identified. However, in real contact tracing data, individuals may be missing from contact networks despite being contacts. As we said in the main text, if there are many missing contacts, our method may have reduced power to detect heterogeneity in susceptibility because our estimates of p_n and p_f may both be biased lower, resulting in a smaller difference between them (S3 Text). This is because individuals that we observe as being exposed once (naive) or twice (focal) but that were actually previously exposed more times may be less likely to be infected than true naive and focal individuals. To assess our method's performance with missing contacts, we simulated infection of individuals that had previous, unobserved exposures (i.e., individuals that were missing contacts in previous contact networks). We first simulated the number of previous exposures ξ for each of F focal individuals and $F(N-1)$ naive individuals. We set $F = 50$, 200, or 1000 and $N = 5$. We assumed that individuals had on average one unobserved previous exposure for illustrative purposes, but this assumption could be easily changed. So, we simulated the number of previous exposures as $\xi \sim \text{Poisson}(1)$. For focal individuals, we added an additional exposure to this to represent the previous exposure that was observed. We then generated a risk of infection $r_{i,\xi}$ for each individual where $r_{i,\xi} \sim \text{Gamma}(k, \theta_\xi)$, $i = 1, ..., FN$. The shape parameter k for the risk distribution does not change over exposures, but the scale parameter θ_ξ does such that $\theta_\xi = \frac{\theta_0}{1+\xi\theta_0}$ where θ_0 is the scale parameter of the risk distribution for individuals that have not been previously exposed (i.e., truly naive individuals). We then simulated the number of naive and focal individuals infected, determined our power to detect heterogeneity in susceptibility, estimated parameters, and predicted disease dynamics the same way as in the main text. For calculating power, we ran $1,000$ simulations. For estimating parameters and predicting disease dynamics, we set $C_c = 1.3$, $E_c = 0.25$, $F = 1000$, $R_{0,c} = 3$, and $\gamma = 0.1$ as in the main text.

With the final simulation of infection, we observed one exposure for naive individuals and two for focal individuals, and there was on average one additional exposure for each individual that we did not capture. Hence, we observed $\frac{1}{2}$ the exposures for naive individuals and $\frac{2}{3}$ the exposures for focal individuals. The total percent of captured exposures depends on the fraction of individuals that are naive versus focal. In our case, 80% of individuals are naive and 20% are focal, so this simulation represents us capturing $\frac{8}{15} \approx 50\%$ of exposures. In other words, this analysis shows the effects of missing approximately 50% of contact events that did not result in infection.

We found that our power to detect heterogeneity in susceptibility is moderately reduced, but we are still able to accurately estimate the level of heterogeneity and predict disease dynamics (Figs A, B, C, D). Our detection power is greatly reduced with $F = 50$ in certain parameter space, but as F increases, the reduction in power is lessened (Figs A, B). So, increasing sample size counteracts the impact of missing contacts. While our parameter estimates do not capture the expected fraction of naive individuals infected E_c (which we will explain does not cause problems), they do accurately capture the level of heterogeneity in susceptibility C_c (Fig C). We estimated a smaller E_c because E_c here is a measure of individuals that are observed to be naive, not individuals that are truly naive. As individuals have been exposed more times than expected, individuals observed to be naive have a lower average susceptibility than truly naive individuals due to the infection selection process. This may seem like a problem for predicting disease dynamics, but it is not. This is because for prediction, we only use $\rho = E_c$ to calculate the contact rate c from an assumed $R_{0,c}$. Otherwise, the disease dynamics depend on C_c. Therefore, we are able to accurately predict disease dynamics with similar 95% CIs to the case where there are not missing contacts (Fig D). Note that we would not expect our 95% CIs to be exactly the same because they are calculated from different datasets.

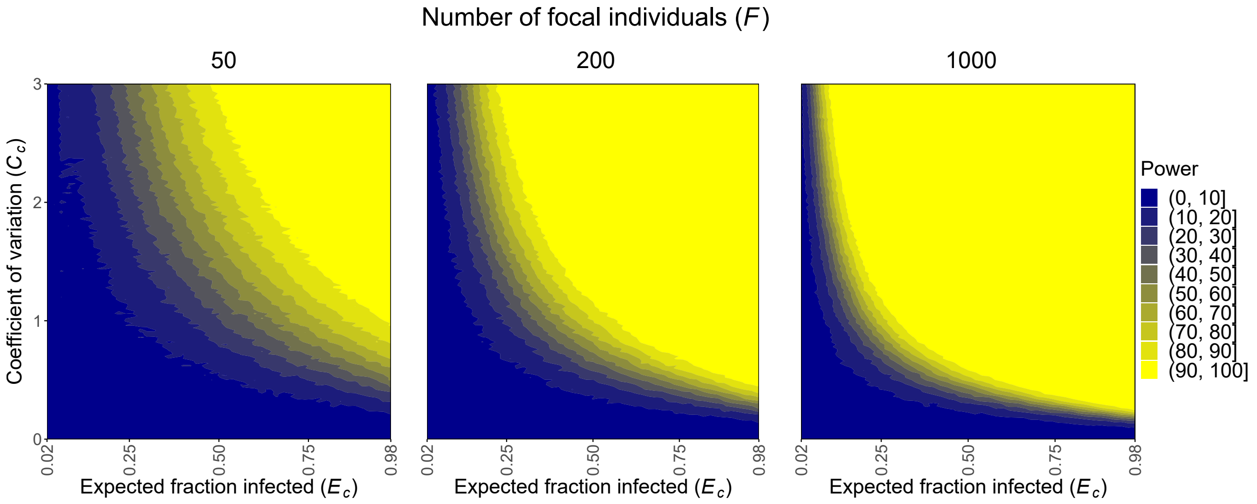

Figure A: The plots show the power to detect heterogeneity in susceptibility in the continuous case with missing contacts, calculated as described in the main text, across different numbers of focal individuals F. These plots can be compared to Figure 4 in the main text to see the effect of missing contacts on detection power, which is also shown below in Figure B. Here only about 50% of exposures are captured. $N = 5$.

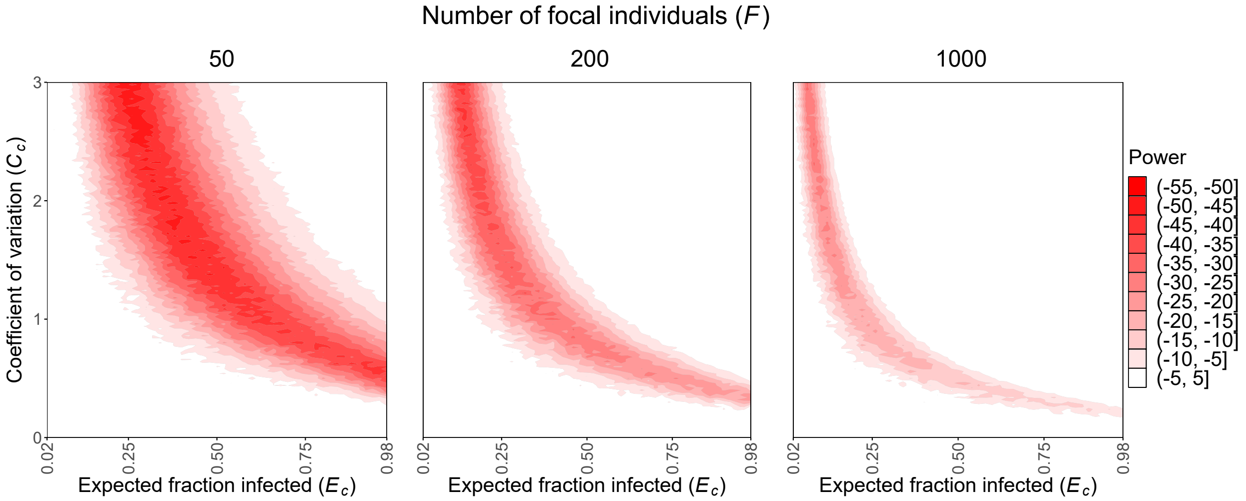

Figure B: The presence of missing contacts reduces our power to detect heterogeneity in susceptibility in the continuous case, but this effect is restricted to the space where heterogeneity is sometimes detectable with or without missing contacts and is lessened with increased sample size. The plots show the difference in the power to detect heterogeneity in susceptibility between the cases where there are and are not missing contacts in the continuous case across different numbers of focal individuals F. This is the difference between Figure A above and Figure 4 in the main text. Negative (red) areas mean that there is less power to detect heterogeneity in susceptibility when there are missing contacts. Here only about 50% of exposures are captured. $N = 5$.

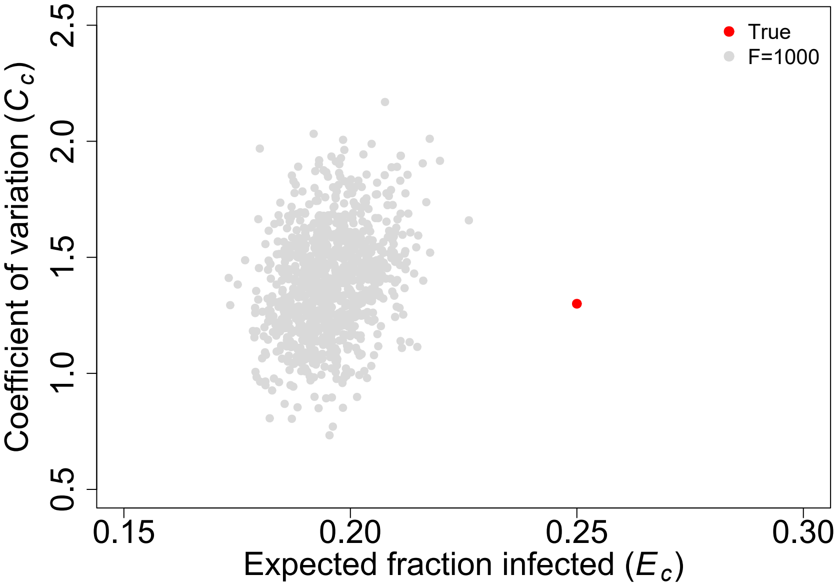

Figure C: With 50% of exposures captured, parameter estimates for the coefficient of variation of risk (C_c) and expected fraction of naive individuals infected (E_c) capture the true value of C_c but not E_c. The plot shows the parameter estimates for C_c and E_c in the continuous case with missing contacts. The red dot represents the true parameters used to generate our simulated data, and the gray dots depict $1,000$ parameter sets from our posterior distribution. Although the parameter estimates do not capture E_c, this is not a problem for predicting disease dynamics because E_c is only used to calculate the contact rate from an assumed $R_{0,c}$. Otherwise, the disease dynamics depend on C_c. $C_c = 1.3$, $E_c = 0.25$, $F = 1000$, and $N = 5$.

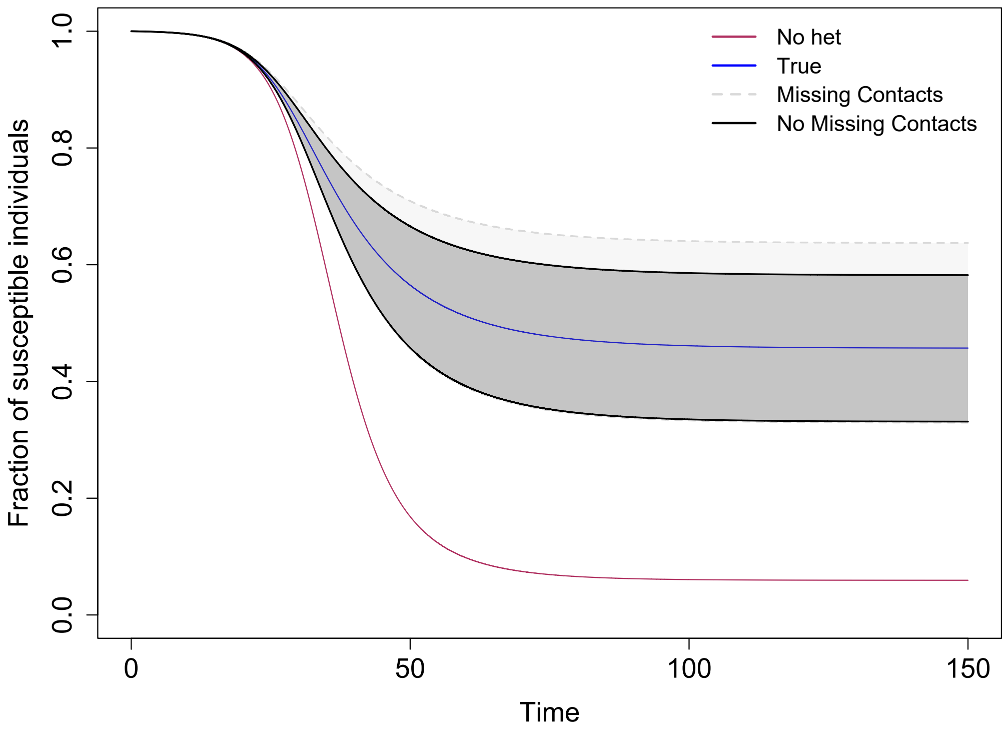

Figure D: With only 50% of exposures captured, the accuracy and precision of our prediction of the disease dynamics is similar to the case where there are not missing contacts. The plots show the effect of missing contacts on predicted SIR dynamics in the continuous case. Specifically, the fraction of susceptible individuals $\frac{S}{S_0}$ is shown over the course of an epidemic. Shaded regions represent 95% CIs determined from 1,000 posterior samples in the case where there are missing contacts (gray) and there are not missing contacts (black). The blue line shows the true dynamics for the parameters used to generate the contact tracing data, and the red line shows the corresponding dynamics if there is homogeneity in susceptibility. $C_c = 1.3$, $E_c = 0.25$, $F = 1000$, and $N = 5$.
